# Human MAIT cells endowed with HBV specificity are cytotoxic and migrate towards HBV-HCC while retaining antimicrobial functions

**DOI:** 10.1016/j.jhepr.2021.100318

**Published:** 2021-06-11

**Authors:** Katie Healy, Andrea Pavesi, Tiphaine Parrot, Michał J. Sobkowiak, Susanne E. Reinsbach, Haleh Davanian, Anthony T. Tan, Soo Aleman, Johan K. Sandberg, Antonio Bertoletti, Margaret Sällberg Chen

**Affiliations:** 1 Department of Dental Medicine, Karolinska Institutet, Stockholm, Sweden; 2 Institute of Molecular and Cell Biology, A∗STAR, Singapore; 3 Center for Infectious Medicine, Karolinska Institutet, Stockholm, Sweden; 4 Department of Biology and Biological Engineering, National Bioinformatics Infrastructure Sweden, Science for Life Laboratory, Chalmers University of Technology, Gothenburg, Sweden; 5 Programme of Emerging Infectious Diseases, Duke-NUS Medical School, Singapore; 6 Department of Infectious Diseases, Karolinska University Hospital, Stockholm, Sweden; 7 Department of Medicine, Karolinska Institutet, Stockholm, Sweden

**Keywords:** HCC, HBV, Adoptive cell transfer, MAIT cells, TCR-T cells, 5-OP-RU, 5-(2-oxopropylideneamino)-6-d-ribitylaminouracil, APC, allophycocyanin, CAR, chimeric antigen receptor, CCR, CC chemokine receptor, ConT, conventional T, CXCL, chemokine (CXC) ligand, CXCR, CXC chemokine receptor, DCI, dead cell index, FMO, fluorescence minus one, FSC, forward scatter, HCC, hepatocellular carcinoma, HLA, human leukocyte antigen, IFN, interferon, IR, irrelevant peptide, MAIT, mucosal-associated invariant T, MFI, mean fluorescence intensity, MHC, major histocompatibility complex, MR1, MHC class I-related molecule, PBMC, peripheral blood mononuclear cell, PE, phycoerythrin, PMA, phorbol myristate acetate, RT, room temperature, SSC, side scatter, TCR, T cell receptor, TNF, tumour necrosis function, UMAP, Uniform Manifold Approximation and Projection, VCAM-1, vascular cell adhesion molecule-1, VLA-4, very late antigen-4

## Abstract

**Background & Aims:**

Virus-specific T cell dysfunction is a common feature of HBV-related hepatocellular carcinoma (HBV-HCC). Conventional T (ConT) cells can be redirected towards viral antigens in HBV-HCC when they express an HBV-specific receptor; however, their efficacy can be impaired by liver-specific physical and metabolic features. Mucosal-associated invariant T (MAIT) cells are the most abundant innate-like T cells in the liver and can elicit potent intrahepatic effector functions. Here, we engineered ConT and MAIT cells to kill HBV expressing hepatoma cells and compared their functional properties.

**Methods:**

Donor-matched ConT and MAIT cells were engineered to express an HBV-specific T cell receptor (TCR). Cytotoxicity and hepatocyte homing potential were investigated using flow cytometry, real-time killing assays, and confocal microscopy in 2D and 3D HBV-HCC cell models. Major histocompatibility complex (MHC) class I-related molecule (MR1)-dependent and MR1-independent activation was evaluated in an *Escherichia coli* THP-1 cell model and by IL-12/IL-18 stimulation, respectively.

**Results:**

HBV TCR-MAIT cells demonstrated polyfunctional properties (CD107a, interferon [IFN] γ, tumour necrosis factor [TNF], and IL-17A) with strong HBV target sensitivity and liver-homing chemokine receptor expression when compared with HBV TCR-ConT cells. TCR-mediated lysis of hepatoma cells was comparable between the cell types and augmented in the presence of inflammation. Coculturing with HBV+ target cells in a 3D microdevice mimicking aspects of the liver microenvironment demonstrated that TCR-MAIT cells migrate readily towards hepatoma targets. Expression of an ectopic TCR did not affect the ability of the MAIT cells to be activated via MR1-presented bacterial antigens or IL-12/IL-18 stimulation.

**Conclusions:**

HBV TCR-MAIT cells demonstrate anti-HBV functions without losing their endogenous antimicrobial mechanisms or hepatotropic features. Our results support future exploitations of MAIT cells for liver-directed immunotherapies.

**Lay summary:**

Chronic HBV infection is a leading cause of liver cancer. T cell receptor (TCR)-engineered T cells are patients’ immune cells that have been modified to recognise virus-infected and/or cancer cells. Herein, we evaluated whether mucosal-associated invariant T cells, a large population of unconventional T cells in the liver, could recognise and kill HBV infected hepatocytes when engineered with an HBV-specific TCR. We show that their effector functions may exceed those of conventional T cells currently used in the clinic, including antimicrobial properties and chemokine receptor profiles better suited for targeting liver tumours.

## Highlights

• Mucosal-associated invariant T (MAIT) cells expanded *in vitro* can be engineered to kill HBV antigen-presenting hepatoma cells.
• MAIT cells produce IL-17A upon encountering their HBV targets, a functional property not observed in conventional T cells currently used in immunotherapy.
• HBV-specific MAIT cells migrate readily towards liver targets in a 3D microfluidics system.
• Co-expression of an HBV-specific TCR does not affect the ability of MAIT cells to respond to their physiological stimuli.

## Graphical abstract

**Figure:**
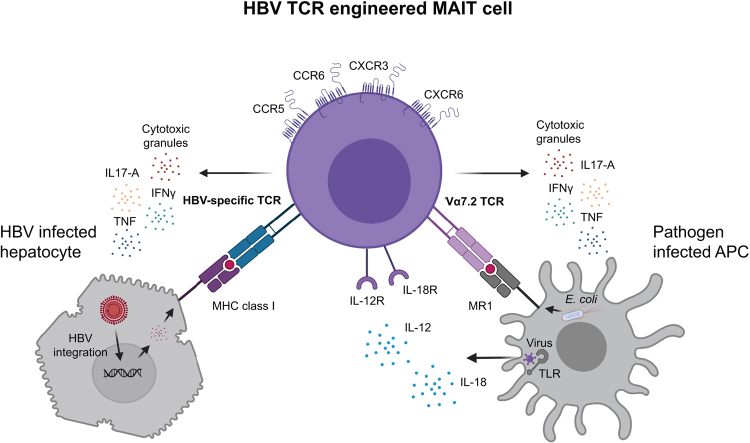

## Introduction

Hepatocellular carcinoma (HCC) is the most common form of liver cancer, and its development is associated with chronic HBV infection.^1^ Many patients with HCC do not present with symptoms until the disease has advanced and spread, resulting in late-stage diagnoses and limited therapeutic options.^2^ Patients with advanced HCC do not typically meet the criteria for surgical and locoregional interventions,^3^ and systemic therapies such as immune checkpoint inhibitors have demonstrated success in only a minority of patients.^4^^,^^5^ Given that reversal of T cell exhaustion alone may not be sufficient to restore host antitumour immunity, adoptive T cell transfer is a promising approach to address this unmet clinical need.

Reprogramming T cells to express T cell receptors (TCRs) with pre-defined specificities has demonstrated success as a cancer treatment,^6^ and its application to infectious diseases is emerging.^7^^,^^8^ As a DNA virus, HBV possesses chromosomal integrative capacity in infected hepatocytes resulting in the production of HBV-human chimeric proteins.^9^^,^^10^ Although this can occur in normal and transformed hepatocytes, the presence of integration events in >90% of HBV-HCC makes viral antigens attractive tumour-associated targets.^11^ Chimeric antigen receptor (CAR)- and TCR-T cells targeting HBV antigens have shown antiviral and anti-HCC functionality *in vitro*^12^^,^^13^ and in murine models of HBV infection.[14], [15], [16] Furthermore, clinical trials investigating their potential under different HBV-associated pathogenic states are ongoing, with preliminary data indicating clinical benefit.^11^^,^^16^^,^^17^

Despite these promising results, cell therapies targeting solid tumours such as HCC are limited by inefficient tumour infiltration and impaired activation within the local immunosuppressive milieu.^18^ Adoptive T cell transfer typically involves systemic infusion, and cross-talk between tumour targets and adoptively transferred cells is crucial to ensure efficient recruitment to the appropriate site.^19^ The trafficking of T cells from the blood to the liver relies on chemokine and cytokine receptor signalling, as well as the upregulation of adhesion molecules and integrins.^18^ It has been described that further engineering of T cells with tumour-specific chemokine receptors can augment their transmigration and antitumoural activity *in vivo*.^20^ However, the introduction of additional transgenes poses a challenge to the manufacturing process as well as the safety of the therapy.

Mucosal-associated invariant T (MAIT) cells are naturally enriched in the liver and represent a critical innate-like T cell subset.^21^^,^^22^ They express a diverse chemokine receptor profile associated with their rapid extravasation and tissue infiltrating properties in response to infection and inflammation.^23^^,^^24^ Unlike conventional T (ConT) cells, which recognise antigens presented by major histocompatibility complex (MHC) class I molecules through highly variable TCRαβ receptors, MAIT cells are MHC class I-related molecule (MR1) restricted and utilise a semi-invariant Vα7.2-Jα33/Jα20/Jα12 TCR with a limited set of β chain diversity. The MAIT cell TCR recognises riboflavin biosynthesis pathway metabolites, earning them a reputation as sentinels of microbial infections.^25^ However, cancer metabolite recognition by MR1-restricted T cells has also been demonstrated,^26^ indicating a role for MR1-dependent host tumour surveillance. TCR-mediated MAIT cell activation results in rapid degranulation and the release of perforin and granzymes, which effectively lyse target cells.^27^^,^^28^ The TCR-activated MAIT cell cytokine profile is a combined Th1/Th17-like phenotype involving interferon (IFN) γ, tumour necrosis factor (TNF), IL-2, IL-22, and IL-17A.^29^ Furthermore, MAIT cells can be activated independently of their TCR; IL-12 and IL-18 can elicit IFNγ production in MAIT cells, and IL-15 can induce innate-like cytotoxic effector functions in liver MAIT cells.^30^

Unlike other human T cell types, MAIT cell potential for liver-targeted immunotherapy is largely unexplored.^31^^,^^32^ Here, we employ a recently established protocol^33^ to investigate TCR-redirected MAIT cells in the context of HBV using a preclinical HCC cell model. Our findings are relevant for the application of TCR-redirected MAIT cells for immunotherapy of liver diseases.

## Materials and methods

### Human samples

For TCR engineering experiments, healthy blood donors were recruited at the Blood Transfusion Clinic at Karolinska University Hospital in Huddinge, Sweden. All donors provided written informed consent under the Declaration of Helsinki and following approval by the Regional Ethics Review Board in Stockholm Dnr 2007/115-31/1. Peripheral blood mononuclear cells (PBMCs) were subsequently isolated by density centrifugation on Ficoll-Paque (Cytiva).

### Donor-matched ConT and MAIT cell expansions

Following isolation from buffy coats, PBMCs were either frozen in 90% FBS + 10% DMSO or underwent MAIT cell expansion as previously described.^33^ On Day 12/14 of the MAIT cell expansion, paired donor PBMCs were thawed and cultured in AIM-V (Thermo Fisher) + 2% AB serum and activated for 7 days in the presence of 50 ng/ml anti-CD3 (eBioscience) and 600 IU/ml IL-2 (Miltenyi) to generate ConT cells as previously reported.^17^ On Day 7, the concentration of IL-2 was increased to 1,000 IU/ml. All cells underwent TCR redirection by electroporation the following day.

### Cytokine functional assays with HBV antigen+ target cells

Human leukocyte antigen (HLA)-A2+ T2 cells (2×10^5^ cells/well) were pulsed in a round-bottomed 96-well plate with HBV_s183-191_ peptide at the concentrations indicated for 1.5 h at 37°C. T2 cells loaded with 1 μg/ml HCV NS3_1073-1081_ peptide served as an irrelevant peptide (IR) control. T2 cells were washed twice and cocultured with TCR-ConT or TCR-MAIT cells at 37°C for 6 h in cRPMI at a 1:1 ratio. HCC cell lines were trypsinised, and 1×10^5^ cells were seeded into a flat-bottomed 96-well plate. Cells were incubated overnight at 37°C to facilitate adherence and washed twice. TCR-redirected ConT or MAIT cells were then added to the targets at a 1:1 ratio in their respective medium and cocultured for 24 h.

### Flow cytometry

To capture intracellular cytokines, protein transport inhibitors GolgiPlug™ and GolgiStop™ (final concentration 1:1,000; BD Biosciences) were added for the final 6 h of coculture assays. The CD107a antibody, where indicated, was added at the time of T cell addition to the target cells. HBV_s183-91_ TCR expression was quantified by staining with an HLA-A2 dextramer (Immudex) loaded with FLLTRILTI peptide at 4°C for 20 min before surface antibody staining. To detect endogenous MAIT cell TCR expression, cells were incubated with either an allophycocyanin (APC) or phycoerythrin (PE)-labelled 5-(2-oxopropylideneamino)-6-d-ribitylaminouracil (5-OP-RU)-loaded tetramer (NIH Tetramer Core Facility) at room temperature (RT) for 40 min before surface antibody staining. The Near-IR Live/Dead Fixable Dead Cell Stain Kit (Thermo Fisher) was used to distinguish live cells in all experiments. For extracellular staining, cells were stained for 20 min in FACS buffer (PBS + 2% FCS + 2 mM EDTA). Stained cells were either fixed in 1× BD CellFix for 10 min at RT or carried forward for intracellular cytokine staining by fixing in BD Fix/Perm (BD Biosciences) for 30 min at 4°C. Cytokine staining was performed in 1× Perm/Wash buffer (BD Biosciences) for 30 min. All antibody stainings were performed at 4°C except for those detecting chemokine receptors, which were performed at RT. All data were acquired on a BD LSRFortessa instrument and analysed using FlowJo software (version 10; BD Biosciences).

### xCELLigence real-time cytotoxicity assay

The HCC adherent cell lines were tryspinised, and 1×10^5^ cells were seeded per well of an xCELLigence E-Plate VIEW 16 (Acea Biosciences). Impedance was monitored at 15-min intervals for 22–24 h using the xCELLigence RTCA DP instrument. For peptide-pulsed conditions, 1 μg/ml HBV_s183-91_ peptide (ProImmune) was added to HepG2 cells for 1.5 h before T cell addition and washed two times in PBS. TCR-redirected ConT or MAIT cells were added at an effector–target 1:1 ratio and monitored continuously for a further 48 h. For inflammation conditions, the cells were cultured in their respective media supplemented with 1,000 IU/ml IFNγ (R&D Systems) and 100 ng/ml TNF (R&D Systems) for the entire duration of the experiment. Killing was calculated as a reduction in the AUC of the effector and target conditions relative to the target only controls for each assay.

### 3D microfluidic assay and image acquisition

The DAX-1 microdevice (AIM Biotech) was used to perform the 3D coculture of the TCR-redirected T cells with the HepG2-PreS1-GFP cells as previously described.[34], [35], [36] Confocal imaging was performed using a 20× objective (numerical aperture 0.38) on the Opera Phenix High-Content Screening System (Perkin Elmer) immediately before and 24 h after addition of the T cells. All imaging was performed at RT, and the cell culture medium in the device served as imaging medium. Acquisition was performed using Perkin Elmer Harmony software. Images were analysed using Imaris x64 (version 9.6.9; Bitplane).

### MAIT cell activation assay

The MAIT cell activation assay was adapted from Dias *et al.*^28^ with minor changes as outlined in the Supplementary information. For IL-12/IL-18 stimulation, TCR-redirected MAIT cells were incubated in the presence of 10 ng/ml IL-12p70 (PeproTech) and 100 ng/ml IL-18 (MBL) for 24 h. THP-1 killing was defined as 100% minus the % of live CD3- cells in the forward scatter/side scatter (FSC/SSC) gate defined in the THP-1 cell-only control. Intracellular cytokines were stained as described above. The MAIT cell activations were performed in cRPMI supplemented with 50 μg/ml gentamicin (Thermo Fisher) and 100 μg/ml Normocin™ (InvivoGen) to prevent contamination by non-fixed bacteria.

### Statistical analysis

Statistical analysis was performed using Prism software (version 8; GraphPad). Datasets initially underwent normality distribution testing. The Wilcoxon signed-rank test and paired *t* tests were used for paired data. The Friedman test with Dunn’s *post hoc* test was performed to detect significance between multiple paired samples. Two-sided *p* values <0.05 were considered significant.

For further details regarding the materials and methods used, please refer to the Supplementary information and CTAT table.

## Results

### Generation and characterisation of donor-matched HBV-specific ConT and MAIT cells

**Fig. 1:**
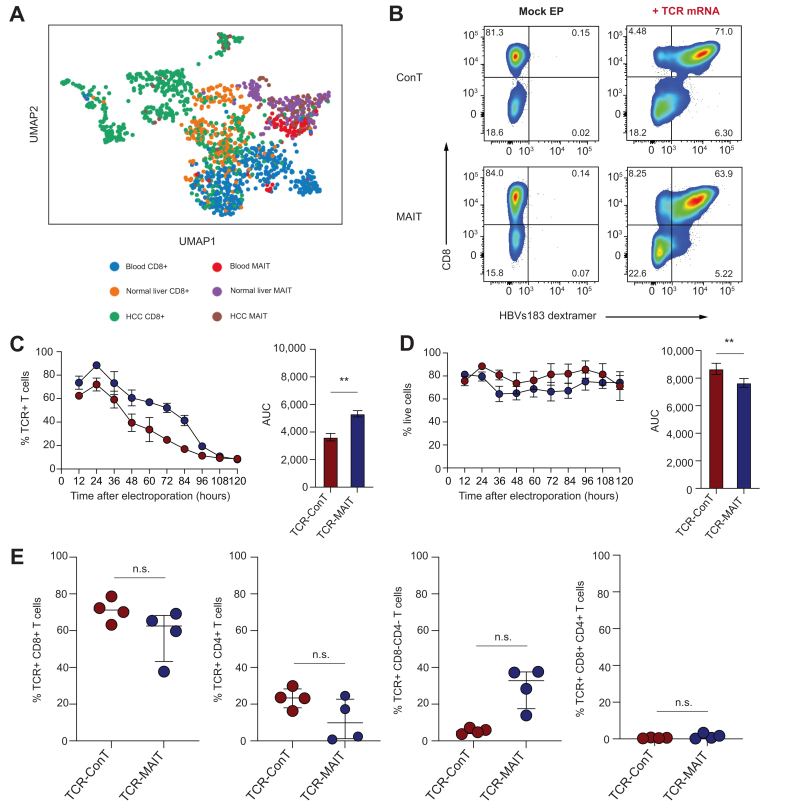
Production and flow cytometric analysis of HBV-specific TCR-ConT and TCR-MAIT cells. (A) UMAP dimension reduction plot of blood, normal liver, and HCC-resident CD8+ and MAIT cell single-cell derived transcriptome datasets. (B) Representative flow cytometry plots from a single donor of HLA-A2-HBV_s183-91_ dextramer staining in HBV_s183-91_ TCR mRNA electroporated ConT and MAIT cells 18 h postelectroporation. Mock electroporated T cells served as negative controls. (C) The HBV TCR expression and (D) cell viability kinetics of transfected ConT and MAIT cells across indicated timepoints and summarized as AUC (n=4 donors). (E) The percentage of CD8+, CD4+, CD8−CD4−, and CD8+CD4+ cells within the HBV TCR+ ConT and MAIT cell populations (n = 4). The lines and error bars represent the mean ± standard error of the mean in (C) and (D), and the median ± IQR in (E). A paired *t* test was performed to detect significance in (C) and (D), and the Wilcoxon matched-pairs signed-rank test was used to detect significance in (E). ∗*p* <0.05, ∗∗*p* <0.01, ∗∗∗*p* <0.001. ConT, conventional T; HCC, hepatocellular carcinoma; HLA, human leukocyte antigen; MAIT, mucosal-associated invariant T cells; n.s., not significant; TCR, T cell receptor; UMAP, Uniform Manifold Approximation and Projection.

To support the rationale for using blood-derived MAIT cells for immunotherapy against liver cancer, we analysed the similarities between T cells residing in both tissue compartments using publicly available single-cell and bulk transcriptome datasets.[37], [38], [39] Conventional CD8+ T cells and MAIT cells derived from patients with HCC were labelled according to their compartment of origin (matched blood, normal liver tissue, and HCC) and analysed by Uniform Manifold Approximation and Projection (UMAP) dimension reduction (Fig. 1A). Unlike the clustering of CD8+ ConT cells, which was highly dependent on the tissue of origin, a strong co-localisation of MAIT cells was observed across all tissues, indicating that blood- and liver-resident MAIT cells share a conserved profile. We reinforced this shared phenotype using an immunological gene expression panel on healthy donor-derived T cells as previously described.^39^ Analysis of the top 50 upregulated genes in each cell type relative to their unstimulated controls following phorbol myristate acetate (PMA)/ionomycin (Fig. S1A) and anti-CD3/CD28-coupled bead (Fig. S1B) stimulation demonstrated that MAIT cells from liver and blood share a similar immunological phenotype following activation. WikiPathway analysis of the genes in the anti-CD3/CD28 condition supported this piece of data, with liver MAIT cells sharing 7 pathways with their blood counterparts, whereas conventional T cells shared 5 pathways (Fig. S1C).

Next, we carried out extensive optimisations of MAIT cell gene transfer using GFP DNA and mRNA reporters (pVAX-enhanced GFP) in a total of 56 conditions, eventually selecting a single condition for the rest of the study (Fig. S2). ConT and MAIT cells were transfected with HBV_s183-91_ TCR mRNA (hereafter referred to as TCR-ConT cells and TCR-MAIT cells, respectively), and surface expression of the TCR was assessed by flow cytometry using an HLA-A2 HBV_s183-91_ dextramer (Fig. 1B). The HBV_s183-91_ TCR genes encode a Vβ3 TCR chain, which can also be used as a marker of HBV TCR expression.^16^ HBV TCR surface expression at 12-h intervals showed similar expression kinetics between the two cell types, with peak expression 24 h after electroporation. Analysis of the AUC of HBV TCR expression in MAIT cells also indicated higher and slightly prolonged TCR expression compared with ConT cells (Fig. 1C). Post-transfection viability staining showed that ConT cells were less prone to transfection-associated cell death than MAIT cells (Fig. 1D). Gating on HBV TCR+ cells did not reveal any significant differences between ConT and MAIT cells on a CD8+, CD4+, double-negative, or double-positive level (Fig. 1E). Taken together, these data demonstrate that blood-derived MAIT cells represent a readily accessible cell source with a liver-tropic phenotype distinct to ConT cells. Furthermore, blood-derived MAIT cells can be engineered with an HBV-specific TCR with high, but transient efficiency and express a CD8+-enriched phenotype in line with cytotoxic effector function.

### Functional assessment of HBV-specific ConT and MAIT cells

**Fig. 2:**
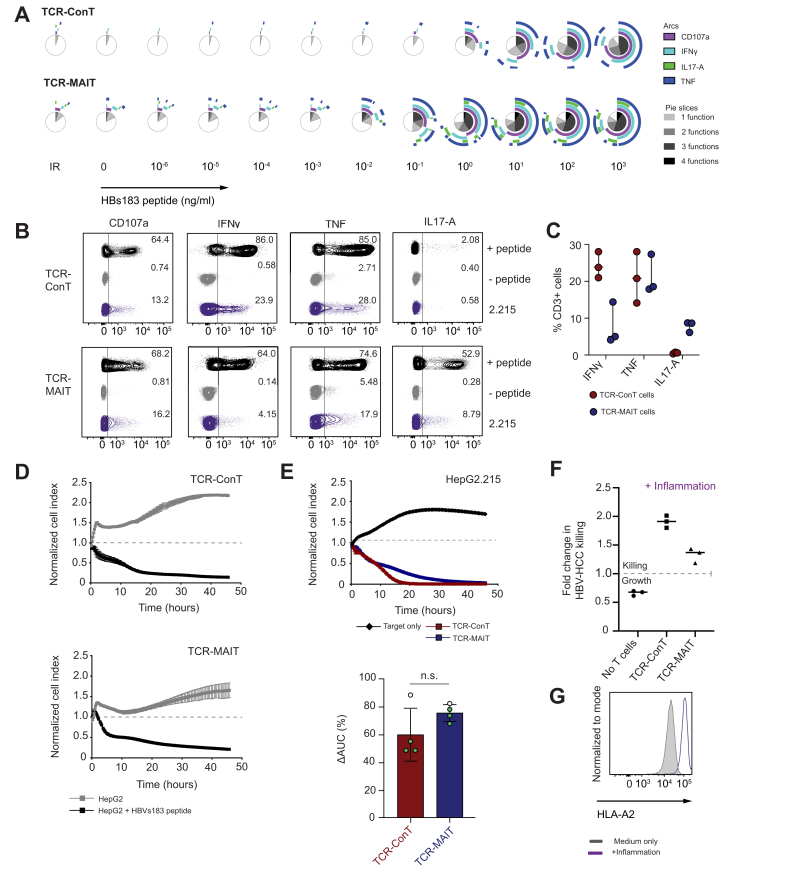
Antiviral and cytolytic capacity of HBV TCR-redirected ConT and MAIT cells. (A) Polyfunctional cytokine profile of TCR-ConT and TCR-MAIT cells following coculture with titrated peptide-loaded T2 cells (n = 1). (B) Representative plots of CD107a, IFNγ, TNF, and IL17-A expression after 12-h coculture with HepG2 cells, HepG2 cells loaded with 1ug/ml HBV peptide, and HepG2.215 cells. (C) Percentages of expression of IFNγ, TNFα, and IL-17A by HBV TCR-redirected ConT and MAIT cells following coculture with the HepG2.2.15 cell line (n = 3 donors). Bars and error bars represent the median and range, respectively. (D) Normalized cell index curves plotting the adherence of HepG2 cells with and without 1μg/ml HBV peptide during coculture with TCR-ConT and TCR-MAIT cells. (E) Representative normalized cell index curve of HepG2.2.15 cells only (black), and cocultured with TCR-ConT (blue) or TCR-MAIT (red) cells. The percentage reduction of the AUC in the presence of TCR-T cells relative to the cell only control is shown. Black dots represent HepG2.215 cells, green dots represent HepG2-PreS1-GFP cells (n = 4 donors). (F) Fold change of HepG2-PreS1-GFP cell lysis following coculture with TCR-ConT and TCR-MAIT cells in the presence of IFNγ and TNF (inflammation). Fold change was calculated as the AUC of the inflammation conditions relative to the AUC of the respective noninflammation conditions (n = 3 donors). The lines and error bars represent the median ± IQR. The Wilcoxon matched-pairs signed-rank test was used to detect significance. ConT, conventional T; HCC, hepatocellular carcinoma; IFN, interferon; IR, irrelevant peptide; MAIT, mucosal-associated invariant T cells; n.s., not significant; TCR, T cell receptor; TNF, tumour necrosis factor.

We next compared the specific T cell recognition and activation profile of HBV TCR-redirected ConT and MAIT cells towards their new target. First, we performed cocultures with HBV_s183-91_ (FLLTRILTI) peptide-loaded T2 cells at antigen concentrations ranging from 10^−6^ to 10^3^ ng/ml. Given that the *ex vivo* expanded MAIT cells have been shown to be CD161 low after expansion,^33^ and that they downregulate CD161 in response to antigenic stimulation, we independently assessed MAIT cell purity after expansion and gated on the total CD3+ populations in both cell preparations for functional assays (Fig. S3). As shown in Fig. 2A, TCR-T cell intracellular cytokine staining and SPICE analysis^40^ after 6 h coculture showed polyfunctional responses down to a 10^−2^ ng/ml level. Although both T cell types upregulated CD107a and produced IFNγ and TNF in an antigen-specific manner, the TCR-MAIT cell polyfunctional profile also included elevated levels of IL-17A. To assess recognition of endogenously processed HBV in the context of HCC cells, we also performed co-cultures with HepG2.215 cells, an HCC cell line constitutively expressing HBV genotype D virus.^41^ TCR-MAIT cells demonstrated ample CD107a and TNF responses to the HepG2.215 cells, and the production of IFNγ by TCR-MAIT cells was not statistically significantly lower than that by TCR-ConT cells. Again, the IL-17A production was restricted to the TCR-MAIT cells only, with TCR-ConT cells remaining IL-17A negative (Fig. 2B,C).

Because the HBV_s183-91_ TCR has previously demonstrated anti-HCC functionality,^11^^,^^16^ we next determined how these MAIT cell responses translated to specific lysis of the target cells. An impedance-based real-time cytotoxicity assay^42^ demonstrated that both cell types were able to effectively lyse FLLTRILTI-loaded HepG2 cells with antigen specificity confirmed by continued HCC cell proliferation in the peptide negative control (Fig. 2D). A similar cytolytic profile was observed in coculture with the HBV antigen-expressing cell lines HepG2.215 and HepG2-PreS1-GFP. Plotting the killing efficiency as the change in the AUC relative to the cell-only controls revealed no significant difference between the cell types. To determine the impact of inflammation on HBV-TCR-mediated killing, IFNγ and TNF were added to the cultures as previously described.^34^ We found that this inflammatory condition enhanced the killing by both cell types to a similar extent (Fig. 2F). IFNγ is known to upregulate surface expression of HLA molecules and therefore enhances antigen presentation to T cells.^11^ We confirmed this in HepG2.215 cells in Fig. 2G, where we observed a log-fold increase in HLA-A2 mean fluorescence intensity (MFI) staining after 24 h culture in the inflammation conditions relative to the unstimulated control (one representative image of three individual experiments). These data demonstrate that the induction of functional and cytotoxic functions in TCR-MAIT cells is rapid, potent, and antigen specific to HBV-associated targets.

### Chemokine receptor and integrin expression profiles in TCR-ConT and TCR-MAIT cells

**Fig. 3:**
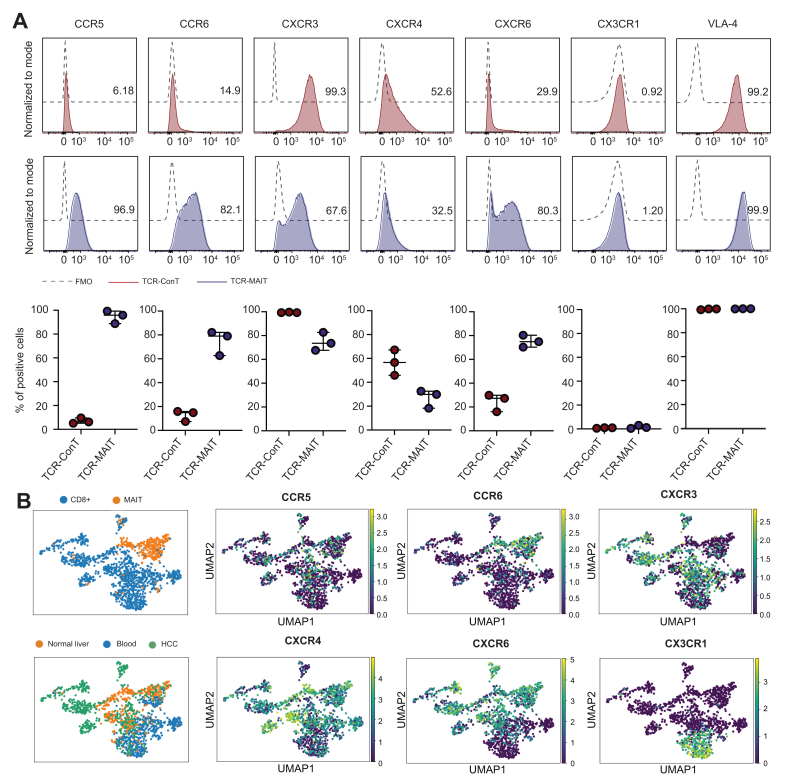
Chemokine receptor, integrin, and activation profile of donor-matched TCR-ConT and MAIT cells. (A) Representative flow cytometry histograms and summary plots of CCR5, CCR6, CXCR3, CXCR4, and CXCR6, CX3CR1, and VLA-4 expression in donor-matched TCR-T cells (n = 3 donors). The FMO controls are shown as black dashed lines. (B) UMAP dimension reduction plots of Leiden clustering by cell type and tissue compartment (left panel). The mean expression of the chemokine receptor transcripts of interest was also plotted using the same community detection algorithm (right panel). The lines and error bars represent the median ± IQR in (A). CCR, CC chemokine receptor; ConT, conventional T; CXCR, CXC chemokine receptor; FMO, fluorescence minus one; HCC, hepatocellular carcinoma; MAIT, mucosal-associated invariant T cells; TCR, T cell receptor; UMAP, Uniform Manifold Approximation and Projection; VLA-4, very late antigen 4.

Chemokine receptors and integrins play a crucial role in recruiting T cells to the liver.^43^ We have recently noted that MAIT cell *ex vivo* expansion enhances their chemokine receptor profile,^33^ however whether the TCR engineering protocol influenced their expression was not known. In line with previous studies, TCR-MAIT cells expressed high levels of CC chemokine receptor (CCR) 5, CCR6, and CXC chemokine receptor (CXCR) 6,^21^ which were little expressed on TCR-ConT cells (Fig. 3A). Moreover, both cell types expressed CXCR3, which is associated with tropism towards IFNγ-inducible ligands and antitumoural responses.^18^ Intermediate expression levels of CXCR4 and shallow expression of CX3CR1 (<2%) were observed in both TCR-T cells. In addition, constitutive expression of the integrin very late antigen-4 (VLA-4) was observed in both cell types, which has been implicated in MAIT cell residency within the liver biliary epithelium.^22^

To assess the abundance of ConT and MAIT cells in patients with HCC, we analysed a publicly available single-cell transcriptome dataset.[37], [38], [39] We generated UMAP dimension reduction plots and visualised the cell type and tissue compartment residency (left panel) together with the tissue distribution of the selected CCRs as shown in Fig. 3B. As noted, the mean CCR5 expression was low overall, with a broad distribution across the clusters of interest. By contrast, CCR6 expression was closely associated with MAIT cells, as well as localisation of normal liver tissue. High CXCR6 expression was associated with ConT and MAIT cells residing in normal liver and HCC. CXCR3 clusters overlapped with CD8+ T cells residing in normal liver and expression was almost completely overlapping with CD8+ T cells in all three tissue compartments.

Taken together, these data show that the chemokine receptor profile of the TCR-MAIT cell is distinct from that of TCR-ConT and favours tropism towards previously described liver-associated chemokines.

### Enhanced liver tropism of TCR-MAIT cells in a 3D model of the liver microenvironment

**Fig. 4:**
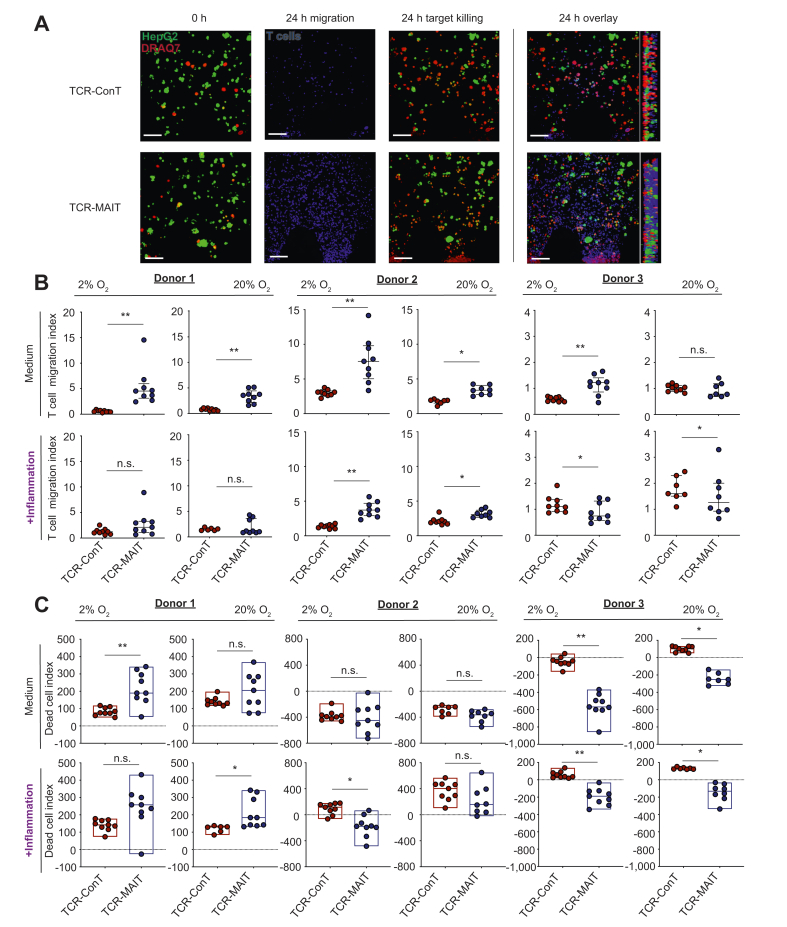
TCR-T cell migration and hepatoma cell killing in a 3D microfluidic device. (A) Representative maximum intensity projection images of HepG2-PreS1-GFP cells (green) in the presence of a DRAQ7 dead cell marker (red) before and after 24 h coculture with TCR-ConT and TCR-MAIT cells. The T cells (blue) were also labelled with a violet cell tracker to quantify their migration towards the hepatoma cells. (B) The migratory properties of the TCR-T cells under different conditions recapitulating the HCC microenvironment was investigated (normoxic and hypoxic cocultures with and without inflammatory cytokines). Collagen gel infiltration is presented as the T cell migration index (the number of T cells that migrate into the device after 24 h coculture normalized to the number of HepG2-preS1 cells within the gel at the time of T cell injection). (C) The killing of HBV Pre-S1-expressing target cells by the TCR-T cells. The TCR normalized DCI was calculated by controlling for differences in TCR transfection efficiency as described. All experiments were performed using 3 healthy donors, and each condition was performed in triplicate. The scale bare represents 100 μm. The lines and error bars represent the median ± IQR. The Wilcoxon matched-pairs signed-rank test was used to detect significance between paired samples. ∗*p* <0.05, ∗∗*p* <0.01, ∗∗∗*p* <0.001. ConT, conventional T; DCI, dead cell index; HCC, hepatocellular carcinoma; MAIT, mucosal-associated invariant T cells; n.s., not significant; TCR, T cell receptor.

The ability of MAIT cells to migrate and kill HBV antigen expressing liver targets was investigated in a 3D microfluidic device, whereby TCR-MAIT and TCR-ConT cells are allowed to interact with hepatoma cells expressing a GFP reporter covalently linked to the HBV envelope protein (HepG2-PreS1-GFP), as previously described.[34], [35], [36] As shown in Fig. 4A, T cells (fluorescently labelled with CellTracker Violet BMQC) loaded in the proximal fluid channel of the device migrate towards hepatoma cells embedded in the 3D gel matrix. The inclusion of a viability dye (DRAQ7) facilitates the quantification of dead target cells at 0 and 24 h after T cell addition. The image analysis pipelines for calculating the T cell migration and dead cell indices are shown in Fig. S4. Cocultures were performed under hypoxic and normoxic conditions to simulate the HCC hypoxic microenvironment. Compared with TCR-ConT, the TCR-MAIT from all three donors migrated significantly more efficiently in both hypoxic (2%) and normoxic (20%) O_2_ concentrations towards the target hepatoma cells, and in the presence of inflammation, this was observed in 1 other donor (Fig. 4B). In 2 of 3 donors (donors 1 and 2), the killing capacity of TCR-MAIT cells appeared similar or higher than that of TCR-ConT cells in both normoxic and hypoxic conditions and also in the presence of inflammation. Conversely, donor 3 TCR-ConT cells outperformed TCR-MAIT cells in these conditions (Fig. 4C). In conclusion, under most conditions, the TCR-MAIT cells migrated more readily towards the hepatoma tumour target, which is in line with their chemokine receptor profile.

### TCR-MAIT cells retain their ability to respond to *Escherichia coli*-fed THP-1 cells and IL-12/IL-18 activation

**Fig. 5:**
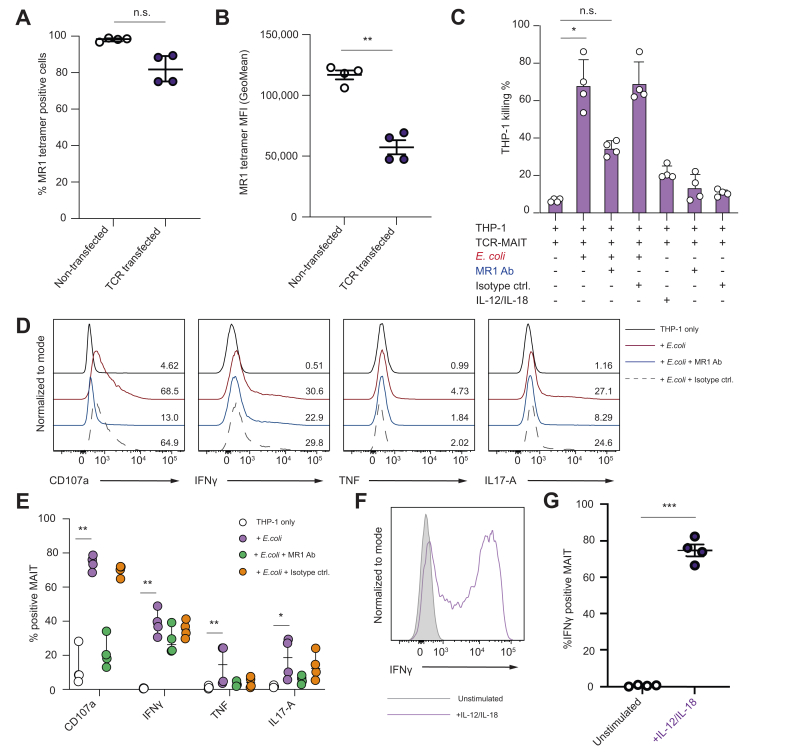
HBV TCR-MAIT cells retain their ability to be activated by MR1-restricted targets and IL-12/IL-18 stimulation. The effect of HBV TCR expression on the percentage (A) and MFI (B) of 5-OP-RU loaded MR1 tetramer binding. (C) Summary plot of THP-1 killing by TCR-MAIT cells following 24 h coculture with non-loaded and *E. coli*-fed THP-1 cells. An MR1 blocking antibody was used in parallel to investigate MR1 dependency on killing. Representative flow cytometry histograms (D) and summary plot (E) of CD107a, IFNγ, TNF, and IL-17A expression under the same conditions. (F) Representative and (G) combined donor data of IFNγ MFI in TCR-MAIT cells with and without stimulation with IL-12/IL-18. The experiment was performed on 4 donors. The lines and error bars represent the median ± IQR in (A), (C), and (E), and the mean ± standard error of the mean in (B) and (G). To detect significance between paired samples, the Wilcoxon matched-pairs signed-rank test was used in (A), and a paired *t* test was performed in (B) and (G). The Friedman test with Dunn’s *post hoc* test was performed to detect significant differences between multiple paired samples in (C) and (E). ∗*p* <0.05, ∗∗*p* <0.01, ∗∗∗*p* <0.001. 5-OP-RU, 5-(2-oxopropylideneamino)-6-d-ribitylaminouracil; IFN, interferon; MAIT, mucosal-associated invariant T; MFI, mean fluorescence intensity; MR1, major histocompatibility complex class I-related molecule; n.s., not significant; TCR, T cell receptor; TNF, tumour necrosis factor.

The functional responses of TCR-MAIT cells through the introduced HBV TCR raised the question of whether they are still capable of exerting their innate-like functions. Co-expression of two TCRs was confirmed by simultaneously staining with a 5-OP-RU-loaded MR1 tetramer and the HBV TCR-specific antibody 24 h after electroporation. As shown in Fig. 5A, the expression of the HBV TCR had a minor impact on the 5-OP-RU-loaded MR1 tetramer binding of TCR-MAIT cells; the vast majority had detectable signal albeit with reduced staining intensity (Fig. 5B). Next, the quality of MR1-dependent or MR1-independent responses were examined by testing TCR-MAIT cells in a THP-1 cell model loaded with *E. coli* (Fig. 5C). TCR-MAIT cells efficiently killed *E. coli* pulsed THP-1 cells relative to controls without bacteria, an effect that was partially dependent on MR1 (blocked by MR1 Ab). Following THP-1 + *E. coli* stimulation, TCR-MAIT cells upregulated CD107a, as well as the proinflammatory cytokines IFNγ, TNF, and IL-17A (Fig. 5D,E). Anti-MR1 blockade further confirmed that CD107a degranulation and cytokine responses were dependent on MR1 as no statistically significant differences were observed between the THP-1 control and the MR1-blockade condition. Furthermore, Fig. 5F,G shows that TCR-MAIT cells were activated by IL-12/IL-18 stimulation, resulting in the production of IFNγ. The latter is attributed to the previously described mechanism by which MAIT cells play a role in antiviral immunity.^44^ These data demonstrate that MAIT cell innate-like functions are well-preserved after TCR redirection and they can recognise multiple antigenic targets through their dual TCR expression.

## Discussion

In this study, we show that *ex vivo* expanded MAIT cells can be redirected towards recognition of HBV antigens through the transfer of virus-specific TCR mRNA. Although MAIT cells are known to exert cytolytic and antiviral effector functions, previous studies have highlighted these properties in the context of innate-like responses during microbial infection. Here, we demonstrated a methodology that can render MAIT cells specific to the vast antigenic potential of MHC class I-restricted targets. This new feature, combined with their unique immunological profile, can be exploited as a therapeutic alternative to ConT cells, which are currently used in immunotherapy. Furthermore, this approach has strong translational potential, as we have recently shown that this method is capable of producing therapeutically relevant numbers of functional MAIT cells for transduction and subsequent transfer (up to 1.9×10^9^ MAIT cells from 50 ml of buffy coat).^33^

Initially, we demonstrated that MAIT cells engineered towards alternative MHC class I-restricted specificities are highly functional, with an array of robust effector functions applicable to HBV immunotherapy. These included polyfunctional cytokine responses, and rapid, potent cytotoxicity, which is in-line with what is known about their effector phenotype.^45^ One distinguishing feature of TCR-MAIT cells was the production of IL-17A upon encountering HBV antigens. MAIT cells are the primary IL-17-producing cell type in the liver,^23^ so this observation was not surprising, although it did constitute a function not seen in ConT cells. The role of IL-17-producing T cells in tumour immunity, however, is still controversial^46^; in mouse models, blocking IL-17^47^ and adoptive transfer of tumour-specific IL-17-producing CD8+ T cells^48^ have both demonstrated antitumoural effects. It has been proposed that the tumour-specific niche within which the IL-17-producing cell resides determines whether IL-17 will be protumorigenic or antitumorigenic.^46^ Therefore, local tumour microenvironment factors, including inflammation status and immune cell composition, could be important for the pathological outcome.

Another difference in the functional profile of both cell types was the differential expression of IFNγ in the TCR-ConT and TCR-MAIT cells following coculture with the HepG2.215 cell line. It is important to note that MAIT cells from the same donor and expansion culture were used for the HBV-associated functional assays as in the *E. coli* and IL-12/IL-18 studies shown later in the manuscript. In the latter, we demonstrated that TCR-MAIT cells were capable of producing large amounts of IFNγ in response to their natural innate targets, confirming that their IFNγ-secreting properties were intact after expansion and redirection. However, when activated by the acquired HLA-restricted TCR, their IFNγ response appeared to be under the influence of the antigen source and peptide magnitude. Indeed, the differential expression of IFNγ by TCR-ConT and IL-17A by TCR-MAIT cells is a feature that may have relevance depending on the downstream applications of the cell product. For example, adoptive transfer of a T cell type that induces a potent proinflammatory response, such as IL-17, may be beneficial from the perspective of tumour elimination, whereas IFNγ production in response to HBV antigens may be more advantageous in the context of viral control. Further investigation of the cytokine profile of TCR-MAIT cells upon encountering low-density targets and following redirection with low-affinity TCRs are also warranted.

In addition to functional and cytotoxic potential, MAIT cell tropism towards the liver formed the basis for their selection for HBV-HCC immunotherapy. We have recently observed that our MAIT cell expansion protocol significantly alters their chemokine receptor profile, driving them towards a unique tissue-homing phenotype.^33^ In line with that study, the TCR-engineered MAIT cells expressed high levels of CCR5, CCR6, CXCR3, CXCR6, and the integrin VLA-4. The recruitment of MAIT cells from the blood has been shown to be associated with the expression of CXCR3 and VLA-4.^22^ In addition to providing physical adhesive support, the binding of VLA-4 to its ligand, VCAM-1, can also support T cell persistence by inhibiting T cell apoptotic signalling.^49^ CXC chemokine ligand (CXCL) 9, CXCL10, and CXCL11 can all be induced by IFNγ, and therefore, an influx of cells that produce IFNγ in response to activation may be of benefit for augmenting T cell migration through the CXCR3 axis.^50^ The tumorigenic process is also known to alter the chemokine production profile of HCC, and screening of the chemokine receptor profile of the cell product, in combination with expression of their cognate ligands within the target tumour, could predict the efficacy of the therapy. To provide more insight into which combinations of chemokine receptors may enhance MAIT cell migration towards HepG2, systematic blocking experiments will be required in future studies.

The 3D microfluidics system used in this study represents a more practical and rigorous evaluation of the TCR-T cells as they also need to actively migrate towards the tumour targets, an interaction that cannot be recapitulated in classical gravity-driven 2D coculture assays.^34^ Under most conditions, we demonstrated that TCR-MAIT cells migrated more readily towards the hepatocytes than did TCR-ConT cells, which aligns with the chemokine receptor phenotype we presented. Interestingly, MAIT cell migration towards the HCC cells was independent of the HBV-TCR expression, with mock-transfected MAIT cells infiltrating the gel region with comparable efficiency (data not shown). Although the 2D culture experiments demonstrated consistent functionality between donors, inter-donor variation was mainly observed in the 3D cocultures. Several different factors may contribute to this variation: chemokine receptor density, hypoxia-inducible factors, and differing immunometabolic status between donors can all modulate the intensity of the stimulus, thereby affecting the magnitude of the downstream signalling pathways. Taken together, the differences observed between donors highlights the sensitivity of this assay, as well as the importance of preclinical testing for predicting the success of an immunotherapy for a specific patient.

We found that the ectopic TCR expression did not prevent TCR-MAIT cells from exerting their intrinsic innate-like properties, indicating that they still support defence against invading pathogens such as *E. coli*. Recent studies indicate that solid tumours are often enriched with intracellular bacteria^51^ and that these intratumoural microbiota can interfere with cancer therapy.^52^ Furthermore, a leaky gut in patients with liver disease can drive MAIT cell dysfunction and bacterial infection susceptibility.^53^ Increased bacterial loads and coinfection of patients already critically ill with chronic viral infection can drive tumour progression. Therefore, the administration of a T cell therapy targeting multiple facets of the tumorigenic process may be of clinical benefit. The various aspects of these functions, for example, responsiveness towards microbiota and stimulation through MR1, remain to be systematically explored in new studies to assess whether they might increase the efficiency of TCR-MAIT cells in killing HepG2 in an antigen-specific manner. MAIT TCR-dependent and MAIT TCR-independent signalling are known to work synergistically during MAIT cell activation,^45^ and whether this effect also occurs in the context of dual TCR expression deserves to be explored. The ability of MAIT cells to be activated by cytokines independently of their TCR is also interesting. Although they may be able to boost TCR-dependent responses, the unpredictability of non-specific activation in off-target sites should be considered prior to clinical translation. MAIT cell therapy could be potentially used as an adjuvant or alternative to other available therapies against advanced HCC or recurrent HCC, such as immune checkpoint inhibition. This is of significance in patients with resistance to immune checkpoint inhibitor therapy, which has poor prognostic outcomes.

However, this study has some limitations. Although this T cell immunotherapy was being investigated in the context of HBV-HCC, all functional experiments were performed using healthy donor PBMCs. Given that MAIT cell functionality during chronic HBV infection^54^ and HCC^37^ is altered, the functional responses observed in this study may not be reflective of patient cell performance. Moreover, the lack of clinical data relating to MAIT cell adoptive transfer means that it is difficult to predict how safe and well tolerated this therapy would be *in vivo*. For example, potential formation of mixed TCR dimers is one safety concern that should be screened by co-staining with MAIT cell TCRα and HBV TCRβ chains in future translational studies. Because the ectopic TCR originally comes from another donor, alloreactivity may also occur. However, the lack of available animal models for HBV-HCC means that rigorous preclinical evaluation is limited. Although studying T cell–tumour cell interactions in a 3D system offers important insights into T cell migratory and functional properties, HCC is a complex tumour to model *in vitro*. Our study did not address fibrotic tissue or MAIT cell interactions with local immune cells, which are important considerations for liver immunotherapy.

Overall, this study provides important novel insights into the potential of human MAIT cells for targeting HCC. However, future studies with patient-derived MAIT cells, as well as the range of non-HBV microbial factors that they can respond to, are required.

## Financial support

This study was supported by grants to MSC from the 10.13039/501100002794Swedish Cancer Society (CAN2016/731, 19 0495 Pj 01 H) and 10.13039/501100004047Karolinska Institutet funding for doctoral students (KID-funding). Additional funds were provided by a KI – Johnson & Johnson Innovation/Janssen grant to J.K.S., M.S.C., and A.B.. K.H. received funding from the 10.13039/501100009747Erik and Edith Fernström Foundation for Medical Research, Sweden. A.P. is supported by the Singapore Ministry of Health’s National Medical Research Council under the Open Fund – Young Investigator Grant (OF-YIRG MOH-OFIRG18nov-0007). S.E.R. is financially supported by the 10.13039/501100004063Knut and Alice Wallenberg Foundation as part of the National Bioinformatics Infrastructure Sweden at SciLifeLab. Further funding to J.K.S. was from the 10.13039/501100004359Swedish Research Council (2016-03052) and the 10.13039/501100002794Swedish Cancer Society (CAN 2017/777). A.B. is supported by the Singapore Ministry of Health’s National Medical Research Council under its Singapore Translational Research Investigator Award (NMRC/STaR/0013/2012).

## Authors’ contributions

Conceived the study design: M.S.C., K.H., J.K.S., A.B. Planned and performed the experiments: K.H., A.P., T.P., M.J.S., A.T.T. Analysed and interpreted the data: K.H., A.P., T.P., S.E.R., H.D., A.T.T., MSC. Contributed clinical material and data interpretation: S.A. Supervised the work: M.S.C., A.B., J.K.S., A.P. Wrote the manuscript: K.H., M.S.C. Reviewed and revised the manuscript critically: all authors.

## Data availability statement

Data supporting this study will be made available upon reasonable request to the corresponding author.

## Conflicts of interest

A.P. is a shareholder and consultant for AIM Biotech Pte Ltd. A.B. and A.T.T. are the Scientific Founder and the Scientific Consultant of Lion TCR Pte. Ltd., respectively, a biotech company developing T cell receptors for treatment of virus-related diseases and cancers. The other authors declare no conflicts of interest that pertain to this work. Please refer to the accompanying ICMJE disclosure forms for further details.
