# Supplementary material for: Human MAIT cells endowed with HBV specificity are cytotoxic and migrate towards HBV-HCC while retaining antimicrobial functions
Source: JHEP Rep. 2021 Jun 11;3(4):100318. doi: 10.1016/j.jhepr.2021.100318 (PMC8327138; doi:10.1016/j.jhepr.2021.100318)
Supplement: Multimedia component 1 [file mmc1.pdf]

Human MAIT cells endowed with HBV specificity are cytotoxic and migrate towards HBV-HCC while retaining antimicrobial functions

Katie Healy, Andrea Pavesi, Tiphaine Parrot, Michał J. Sobkowiak, Susanne E. Reinsbach, Haleh Davanian, Anthony T. Tan, Soo Aleman, Johan K. Sandberg, Antonio Bertoletti, Margaret Sällberg Chen

Table of contents

Supplementary materials and methods……………………………………………………2

Supplementary figures…………………………………………………………...............8

Supplementary references…………………………………………………………….…..13

Supplementary material and methods

Cell lines. T2, HepG2-PreS1-GFP, and THP-1 cells were cultured in complete RPMI (cRPMI) culture media (RPMI 1640 supplemented with 10% heat-inactivated FBS, 20 mM HEPES, 0.5 mM sodium pyruvate, 100 IU/ml penicillin, 100 µg/ml streptomycin, MEM nonessential amino acids (Sigma)). HepG2 and HepG.2.215 cells were cultured in D10 medium (DMEM supplemented with 10% heat-inactivated FBS, 100 IU/ml penicillin, and 100 µg/ml streptomycin (Sigma)). HepG2-PreS1-GFP and HepG2.2.15 cells were also cultured in the presence of 5 µg/ml puromycin (Fisher Scientific) and 100 µg/ml geneticin™ (G418 sulfate; Gibco), respectively, to facilitate the culture of transgene expressing cells only.

Single cell gene expression analysis. Single cell RNA-seq data on human HCC[1] was downloaded from Gene Expression Omnibus (GEO accession GSE98638). We analyzed the pre-processed gene expression data from single T cells isolated from peripheral blood, tumor and normal tissue of five patients. The classification of cells as MAIT cells was provided by Yao et al.[2] and we analyzed only cells in which at least one productive TCRαβ was identified according to the results of the original article. scRNA-seq data were analyzed using the SCANPY toolkit for preprocessing, visualization, clustering and differential testing.[3] In brief, basic filtering was applied to remove genes and cells below a minimum threshold (min_genes=300, min_cells=10). A library-size correction was performed by normalizing the data to 10,000 reads per cell and only highly variable cells (min_mean=0.0125, max_mean=3, min_disp=0.5) were kept for further analysis. Next, a principle component analysis was applied and the top 30 principle components were used for the neighborhood graph computation (n_neighbors=7, n_pcs=30, metric="euclidean"). The cells were clustered using the Leiden algorithm for community detection. A total of 15 clusters were identified and

visualized in the Uniform Manifold Approximation and Projection (UMAP) embedding. In addition, the UMAP embedding was color-coded by the cell type and source. Differential gene expression was performed using Wilcoxon rank-sum (Mann-Whitney-U) test comparing MAIT and CD8+ T cells in the respective tissues.

Human samples for immunological profiling. For gene expression analyses on healthy donors, the study was approved by the Gleneagles Hospital Ethics Committee, Singapore, and all participants gave written informed consent. Liver-associated mononuclear cells were collected after portal flush using preservation solution at 4°C according to standard protocol preceding liver transplantation.[4] Blood samples were then collected from age-matched healthy controls. All cells were subsequently isolated by density centrifugation on Ficoll-Paque (GE Healthcare).

Cell sorting and multiplex gene expression analysis. The sorting, stimulation, and intial gene expression analysis of this dataset has been previously described.[5] Briefly, MAIT cells were sorted based on $CD3^{+}CD161^{high}V\alpha 7.2^{+}$ expression. Conventional T cells were extracted based on $CD8^{+}$ staining from the MAIT negative fraction and were confirmed to co-express $CD3^{+}$ and not $CD161^{high}V\alpha 7.2^{+}$. Stimulation was performed by culturing the cells overnight in the presence of either PMA (2 ng/ml) and ionomycin (1 µg/ml) or anti-CD3/CD28-coupled beads (1:1 bead:cell ratio; Invitrogen). The sorted cells were lysed and processed using the nCounter GX Human Immunology Kit before analysis in the nCounter ® instrument (Nanostring Technologies), according to the manufacturer's instructions. To remove background non-specific probe binding (noise), a cut-off of two times the mean of the negative control samples supplied in the kit was applied. The samples were then normalized based on the geometric mean of the housekeeping gene panel and positive controls supplied in the kit. The coefficient of variation (SD of the normalized

counts across all samples/mean normalized counts across all samples, expressed as a percentage) of the housekeeping genes was calculated and used as a cut-off to filter out genes that remained stable across the samples. For WikiPathway enrichment analysis the WEB-based Gene Set Analysis Toolkit version 3 (WebGestalt) was used (REF PMID: 31114916). The following parameters were chosen: ID type: ENSEMBL protein ID, Reference set: Genome, Statistical method: hypergeometric, multiple test adjustment: Benjamini & Hochberg (BH). Significance level was set to FDR <0.05 and minimum number of five and maximum number of 2000 genes for each pathway. The top 10 pathways are presented.

MAIT cell isolation and expansion. MAIT cells were labelled with an APC or PE-conjugated 5-OP-RU MR1-restricted tetramer (NIH Tetramer Core Facility) for 10 min at 4°C, followed by MACS anti-APC or anti-PE microbead magnetic separation (Miltenyi) according to the manufacturer's instructions. The negative PBMC fraction was irradiated at 35 Gray, and resuspended at $2x10^6$ cells/ml in MAIT cell medium (Immunocult™-XF T cell Expansion Medium (STEMCELL)), 8% CTS™ Immune Cell Serum Replacement (Thermofisher), 100 U/ml, 100 µg/ml penicillin/streptomycin (Sigma), 100 ug/ml Normocin™ (Invivogen)) and 100 µl of the suspension was plated in 96-well U-bottomed plates. The purified MAIT cells were resuspended at $4x10^5$ cells/ml and 50 µl was added on top of the irradiated PBMCs. The MAIT cells were cultured in 50 ng/ml animal-free recombinant IL-2 (PeproTech), and were either split or fed with fresh cytokine every 2-3 days for 19-21 days before electroporation.

TCR mRNA synthesis. DNA encoding $HBV_{s183-91}$ epitope-specific TCR genes was subcloned into the pVAX1 vector as previously described.[6] The plasmid was linearized by restriction digest using the XbaI enzyme (Thermofisher). mRNA was transcribed in vitro using the mMessage mMachine™ T7 ULTRA Transcription kit (Invitrogen)

according to the manufacturer's instructions. mRNA was precipitated in lithium chloride solution, resuspended in nuclease-free water at 2 µg/ml, and stored at -80°C until use.

Electroporation of MAIT and ConT cells. For MAIT transfection optimization assays, the Cell Line Optimization 4D-Nucleofector™ X Kit (Lonza) was used. On day 19/21 of MAIT expansion, the MAIT cells were resuspended at 100 $x10^6$/ml in either SE, SF, or SG nucleofector™ X solution. The GFP nucleic acids were added to the solutions and 20 µl of cell suspension was added to each well of a 16-well Nucleocuvette strip (2 $x10^6$ cells + 0.5 µg GFP DNA/4 µg mRNA per well). Each well received a pulse according to the pre-defined optimization settings using the 4D Nucleofector X Unit. For electroporation using TCR mRNA, $10x10^6$ MAIT cells were suspended in 100µl supplemented SF Nucleofector™ solution (Lonza) with 200 µg/ml TCR mRNA. The mixture was placed in a Nucleocuvette™ vessel (Lonza) and pulsed using the EN-138 pulse setting. For ConT cell electroporation with TCR mRNA, $10x10^6$ ConT cells were suspended in 100µl supplemented Cell Line Nucleofector™ Kit V solution (Lonza) with 200 µg/ml TCR mRNA. The mixture was placed in a 100 µl aluminium cuvette (Lonza) and pulsed using the X-01 pulse setting on the Amaxa 2b (Lonza).

After electroporation, the T cells were cultured in their respective media at 37°C and 5% CO_2 until analysis. All experiments involving TCR-T cell functionality and phenotyping were performed 18-24 h after electroporation unless otherwise stated.

Flow cytometry. The antibodies used for staining are further specified in the supplementary CTAT table: CD3-BV650 clone OKT3, CD8-BV570 clone RPA-T8, CD4-BV711 clone OKT4, CD161-PECy5 clone DX12, Vα7.2-APC clone 3C10, VB3-FITC clone CH92, IL17A-BV421 clone BL168, IFNγ-BV785 clone 4SB34, TNF-PECy7 clone MAb11, CD107a-BUV395, CD3-FITC clone UCHT1, Vα7.2-PE clone 3C10,

CCR5-BUV395 clone 2D7, CCR6-BV650 clone G034E3, CXCR3-PECy7 clone G025H7, CXCR4-BV785 clone L276F12, CXCR6-AF647 clone K041E5, CX3CR1-BV421 clone 2A9-1, CD49d-APC clone 9F10, and HLA-A2-FITC clone BB7.2.

3D microfluidic assay. To prepare 100 µl of collagen gel solution containing dissociated hepatoma cells, 10 µl 10X PBS, pH 7.4 (Life Technologies) containing phenol red sodium salt (Sigma) was mixed with 71 µl collagen type-1 (rat tail; Corning), 2.4 µl NaOH, 6.6 µl Water For Injection (Life Technologies), and 10 µl of freshly trypsinized HepG2 cells at $25x10^6$cells/ml. The final pH of the collagen/cell suspension was confirmed as close to 7 with litmus paper (Sigma), and 10 µl was injected into the gel entry port of each device. The gel was polymerized by incubating at 37°C for 30 min and then hydrated by bringing the medium channels to a final volume of 270 µl cRPMI. The viability marker DRAQ7 (Miltenyi) was also mixed with the R10 at a concentration of 3µM to distinguish dead cells, and the devices were placed in the incubator at 37°C and 5% CO_2. After 24 h, the R10 was aspirated off and the devices containing ConT and MAIT cells were flushed with ConT medium supplemented with 100 IU/ml IL-2 (Miltenyi) and MAIT medium supplemented with 50 ng/ml IL-2 (PeproTech), respectively. DRAQ7 was again added at 3µM for live/dead discrimination. To visualize the T cells, they were stained in serum-free RPMI 1640 containing 3 µM Cell Tracker Violet BMQC dye (Thermofisher Scientific) for 30 min at 37°C. Stained ConT and MAIT cells were washed and resuspended in their respective media and incubated for a further 30 min at 37°C, before being resuspended to a final concentration of $3x10^6$/ml. The T cell suspension (30 µl) was then added to one of the media channels adjacent to the collagen gel. Finally, the co-cultured cells were incubated for 24 h under either normoxic (20% O_2) or hypoxic (2% O_2) conditions.

3D image analysis. Imaris blended mode was used to generate representative images, while maximum intensity projections of Z-stacks were used for the quantitative analysis. Autoflourescence from support posts within the microfluidic chip was removed from the representative images using Adobe Photoshop. The T cell migration index was calculated as the number of T cells that migrated into a region of the microfluidic device relative to the number of live hepatoma cells at the time of injection. A Dead Cell Index (DCI) was used to quantify hepatoma cell lysis, where the number of dead targets was normalized to TCR expression to account for differences in transfection efficiency as previously described.[7] Each condition was tested in triplicate and 3 regions were imaged per replicate.

MR1-dependent activation of TCR-redirected MAIT cells. THP-1 cells were plated at $1x10^5$ cells per well in a 96-well U-bottomed plate and incubated for 1.5 h. The *E. coli* (strain D21) was thawed on ice, washed with PBS, and fixed for 3 min in 1X BD Cellfix (BD Biosciences), with intermittent vortexing. The bacteria were washed repeatedly with 1X PBS and incubated with the THP-1 cells for 3 h (MOI = 30). For MR1 blocking experiments, an anti-MR1 antibody (26.5; Biolegend), or isotype control (MOPC-173; Biolegend) was added at a final concentration of 20 µg/ml. TCR-redirected MAIT cells (18 h after electroporation) were added to the *E. coli* fed THP-1 at a 1:1 ratio in the presence of a CD107a antibody (BD Biosciences). After 1 h, an anti-CD28 antibody (L293; BD) at 1.25 ng/ml was added and the cells were cultured for 24 h.

Fig. S1. Gene expression analysis of healthy donor liver MAIT cells and blood-derived CD8+ T cells and MAIT cells.

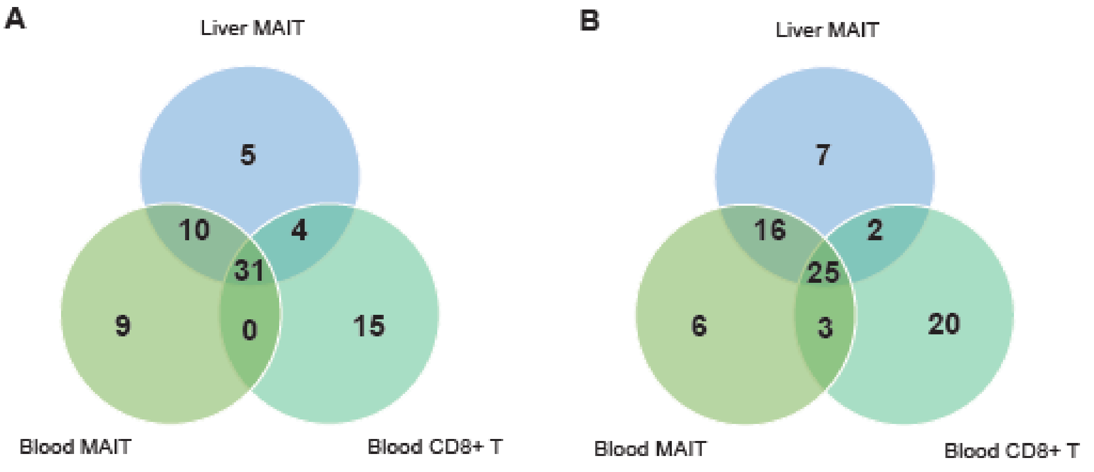

C

Liver MAIT

Pathway	p-value	FDR
1. T cell antigen receptor pathway during *Staphylococcus aureus* infection	8.6361e-11	3.2904e-8
2. Apoptosis	1.7628e-9	3.0541e-7
3. Allograft rejection	2.4048e-9	3.0541e-7
4. miRNAs involvement in the immune response in sepsis	3.2720e-9	3.1166e-7
5. Inflammatory response pathway	3.8924e-8	0.0000029660
6. EBV LMP1 signaling	2.8423e-7	0.000018049
7. Cytokines and Inflammatory response	4.3589e-7	0.000023725
8. T cell receptor and co-stimulatory signaling	7.7626e-7	0.000036969
9. Photodynamic and therapy-induced NF-κB survival signaling	0.0000020648	0.000087411
10. Regulation of toll-like receptor signaling pathway	0.0000023326	0.000088870

Blood MAIT

Pathway	p-value	FDR
1. T cell antigen receptor pathway during *Staphylococcus aureus* infection	2.9423e-12	1.1210e-9
2. Allograft rejection	3.0671e-9	5.8429e-7
3. IL-2 signaling pathway	5.8422e-9	7.4196e-7
4. T cell antigen receptor pathway and co-stimulatory signaling	1.9937e-8	0.0000018990
5. IL-4 signaling pathway	4.1365e-8	0.0000031520
6. Apoptosis	5.0016e-8	0.0000031760
7. miRNAs involvement in the immune response in sepsis	1.0866e-7	0.0000059140
8. Regulation of toll-like receptor signaling pathway	2.2691e-7	0.000010806
9. EBV LMP1 signaling	3.2401e-7	0.000013716
10. Regulation of toll-like receptor signaling pathway	4.0411e-7	0.000014346

Blood CD8+ T

Pathway	p-value	FDR
1. Cytokines and Inflammatory response	2.5535e-14	9.7289e-12
2. Development and heterogeneity of the ILC family	1.5731e-11	2.9967e-9
3. Allograft rejection	1.6147e-10	2.0507e-8
4. Type II interferon signaling (IFNG)	2.7402e-9	2.6100e-7
5. Development of pulmonary dendritic cells and macrophage subsets	0.0000010964	0.000083548
6. T cell antigen receptor pathway during *Staphylococcus aureus* infection	0.0000025692	0.00015381
7. Lung fibrosis	0.0000028259	0.000023725
8. Regulation of toll-like receptor signaling pathway	0.000036558	0.0017411
9. miRNAs involvement in the immune response in sepsis	0.000050438	0.0019217
10. Hematopoietic stem cell differentiation	0.000050438	0.0019217

Venn diagrams showing the distribution of shared genes among liver MAIT cells and blood-derived CD8+ T cells and MAIT cells following stimulation with PMA/ionomycin (A) and anti-CD3/CD38-coupled beads (B). (C) WikiPathway enrichment analysis of the top 10 intracellular pathways regulated among liver MAIT cells and blood-derived CD8+ T cells and MAIT cells following stimulation with anti-CD3/CD38-coupled beads (n=4; two donors from each compartment).

Fig. S2. Ex vivo expanded MAIT cell transfection optimization.

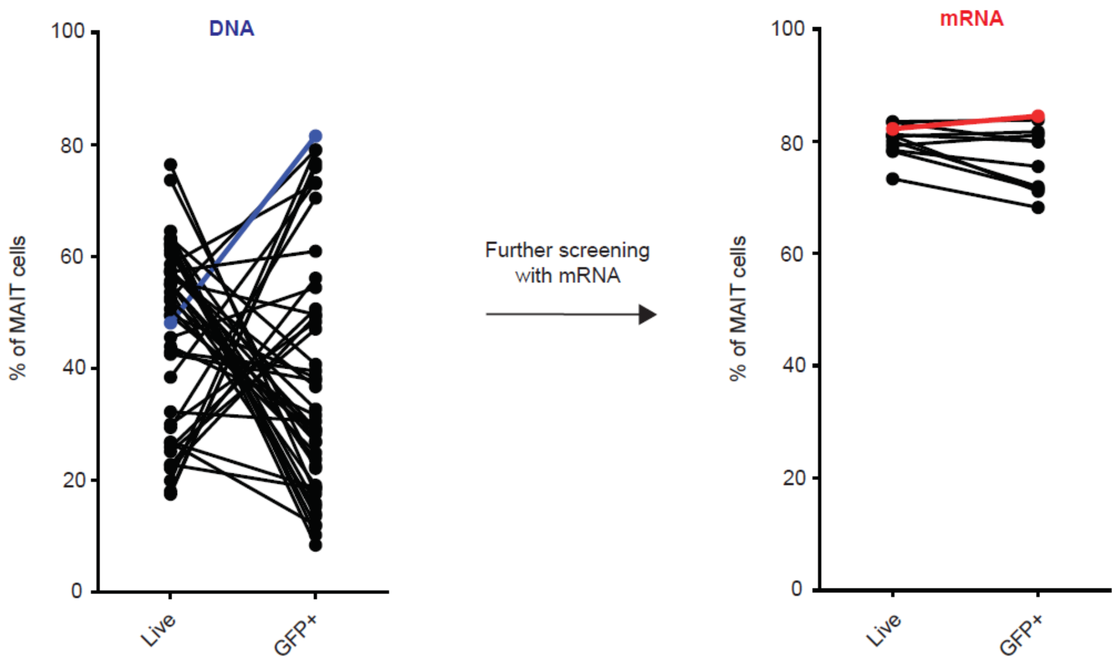

Optimization of the MAIT cell transfection protocol was initially performed using GFP DNA (45 conditions; 15 pulse settings in combination with three transfection solutions). One condition giving favorable gene expression was carried forward for analysis with GFP mRNA using 11 similar pulse-settiings. The condition highlighted in red was used for subsequent mRNA transfections presented in the study.

Fig. S3. Gating strategy for TCR-T cell functional assays.

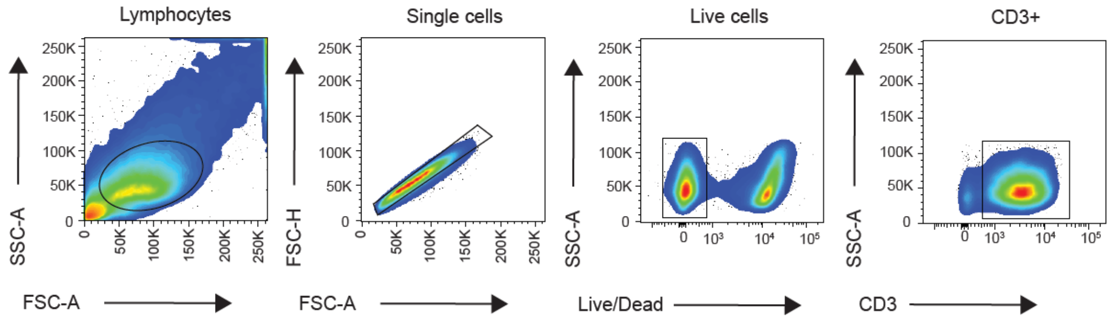

The gating strategy for the flow cytometric analysis of TCR-redirected ConT and MAIT cells in functional assays is presented.

Fig. S4. Image analysis pipelines for 3D co-culture assays.

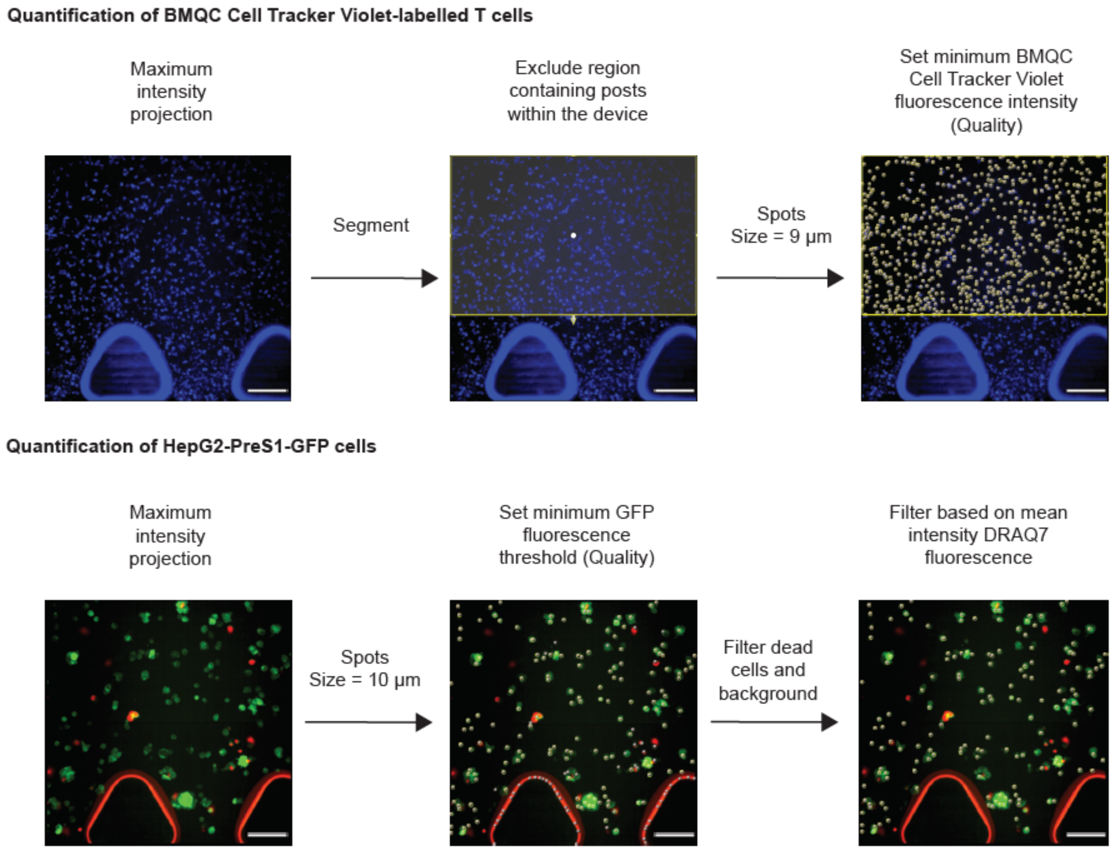

Maximum intensity Z-projections of the microfluidic device were used for quantitative analyses. For T cell quantification, the images were segmented to remove intereference by the support posts within the device. The ‘Spots’ function was used to count the number of T cells (size = 9 μm) within the highlighted region based on a minimum value for signal intensity at the center of the spots (Quality; upper panel). To calculate the number of live HepG2-PreS1-GFP cells within each image, the ‘Spots’ function was applied to detect objects within the GFP channel (size = 10 μm) using ‘Quality’ to set a minimum GFP fluorescence threshold. A filter based on the mean intensity of DRAQ7 fluorescence was subsequently used to remove dead cells and background from the support posts (lower panel). The scale bar represents 100 μm.

Supplementary references

Author names in bold designate shared co-first authorship

1. Zheng C, Zheng L, Yoo JK, Guo H, Zhang Y, Guo X, et al. Landscape of Infiltrating T Cells in Liver Cancer Revealed by Single-Cell Sequencing. Cell. 2017;169(7):1342-1356.e1316.
2. Yao T, Shooshtari P, Haeryfar SMM. Leveraging Public Single-Cell and Bulk Transcriptomic Datasets to Delineate MAIT Cell Roles and Phenotypic Characteristics in Human Malignancies. Frontiers in Immunology. 2020;11(1691).
3. Wolf FA, Angerer P, Theis FJ. SCANPY: large-scale single-cell gene expression data analysis. Genome Biology. 2018;19(1):15.
4. Tu Z, Bozorgzadeh A, Crispe IN, Orloff MS. The activation state of human intrahepatic lymphocytes. Clin Exp Immunol. 2007;149(1):186-193.
5. Tang XZ, Jo J, Tan AT, Sandalova E, Chia A, Tan KC, et al. IL-7 licenses activation of human liver intrasinusoidal mucosal-associated invariant T cells. J Immunol. 2013;190(7):3142-3152.
6. Koh S, Shimasaki N, Suwanarusk R, Ho ZZ, Chia A, Banu N, et al. A practical approach to immunotherapy of hepatocellular carcinoma using T cells redirected against hepatitis B virus. Mol Ther Nucleic Acids. 2013;2(8):e114.
7. **Lee SWL**, **Adriani G**, **Ceccarello E**, Pavesi A, Tan AT, Bertoletti A, et al. Characterizing the Role of Monocytes in T Cell Cancer Immunotherapy Using a 3D Microfluidic Model. Frontiers in Immunology. 2018;9(416).
