# Supplementary material for: Human MAIT cells endowed with HBV specificity are cytotoxic and migrate towards HBV-HCC while retaining antimicrobial functions
Source: JHEP Rep. 2021 Jun 11;3(4):100318. doi: 10.1016/j.jhepr.2021.100318 (PMC8327138; doi:10.1016/j.jhepr.2021.100318)
Supplement: Multimedia component 2 [file mmc2.docx]

**JHEP Reports**

**CTAT methods**

Tables for a “Complete, Transparent, Accurate and Timely account” (CTAT) are now mandatory for all revised submissions. The aim is to enhance the reproducibility of methods.

- Only include the parts relevant to your study
- Refer to the CTAT in the main text as ‘Supplementary CTAT Table’
- Do not add subheadings
- Add as many rows as needed to include all information
- Only include one item per row

**If the CTAT form is not relevant to your study, please outline the reasons why:**

|  |
| --- |

- 1. **Antibodies**

| **Name** | **Citation** | **Supplier** | **Cat no.** | **Clone no.** |
| --- | --- | --- | --- | --- |
| CD3 Monoclonal Antibody |  | ThermoFisher Scientific | 16-0037-81 | OKT3 |
| Purified Mouse Anti-Human CD28 |  | BD Biosciences | 348040 | L293 |
| Purified anti-human/mouse/rat MR1 Antibody |  | Biolegend | 361102 | 26.5 |
| Purified Mouse IgG2a, κ Isotype Ctrl Antibody |  | Biolegend | 400201 | MOPC-173 |
| Brilliant Violet 650™ anti-human CD3 Antibody |  | Biolegend | 317323 | OKT3 |
| FITC Mouse Anti-Human CD3 |  | BD Pharmingen™ | 555332 | UCHT-1 |
| Brilliant Violet 570™ anti-human CD8a Antibody |  | Biolegend | 301037 | RPA-T8 |
| Brilliant Violet 711™ anti-human CD4 Antibody |  | Biolegend | 317439 | OKT4 |
| PE-Cy™5 Mouse Anti-Human CD161 |  | BD Biosciences | 551138 | DX12 |
| APC anti-human TCR Vα7.2 Antibody |  | Biolegend | 351707 | 3C10 |
| PE anti-human TCR Vα7.2 Antibody |  | Biolegend | 351705 | 3C10 |
| TCR Vβ3-FITC |  | Beckman Coulter | IM2372 | CH92 |
| BUV395 Mouse Anti-Human CD107a |  | BD Biosciences | 565113 | H4A3 |
| Brilliant Violet 785™ anti-human IFN-γ Antibody |  | Biolegend | 502541 | 4S.B3 |
| PE-Cy™7 Mouse Anti-Human TNF |  | BD Biosciences | 557647 | Mab11 |
| Brilliant Violet 421™ anti-human IL-17A Antibody |  | Biolegend | 512321 | BL168 |
| BUV395 Mouse Anti-Human CD195 |  | BD Biosciences | 565224 | 2D7/CCR5 |
| Brilliant Violet 650™ anti-human CD196 (CCR6) Antibody |  | Biolegend | 353425 | G034E3 |
| PE/Cyanine7 anti-human CD183 (CXCR3) Antibody |  | Biolegend | 323719 | G025H7 |
| Brilliant Violet 421™ anti-mouse CD184 (CXCR4) Antibody |  | Biolegend | 146511 | L276F12 |
| Alexa Fluor® 647 anti-human CD186 (CXCR6) Antibody |  | Biolegend | 356007 | K041E5 |
| Brilliant Violet 421™ anti-human CX3CR1 Antibody |  | Biolegend | 341619 | 2A9-1 |
| APC anti-human CD49d Antibody |  | Biolegend | 304307 | 9F10 |
| FITC Mouse Anti-Human HLA-A2 |  | BD Biosciences | 551285 | BB7.2 |

- 1. **Cell lines**

| **Name** | **Citation** | **Supplier** | **Cat no.** | **Passage no.** | **Authentication test method** |
| --- | --- | --- | --- | --- | --- |
| T2 |  | ATCC | ATCC® CRL-1992™ |  |  |
| HepG2 | DOI: 10.1053/j.gastro.2019.01.251 |  |  |  |  |
| HepG1-preS1-GFP | DOI: 10.1172/jci.insight.89762 |  |  |  |  |
| HepG2.215 | DOI: 10.1053/j.gastro.2019.01.251 |  |  |  |  |
| THP-1 |  | ATCC | ATCC® TIB-202™ |  |  |

- 1. **Organisms**

| **Name** | **Citation** | **Supplier** | **Strain** | **Sex** | **Age** | **Overall n number** |
| --- | --- | --- | --- | --- | --- | --- |
| *E. coli* | DOI: 10.1017/s0016672300011782 | CGSG | D21 |  |  |  |

- 1. **Sequence based reagents**

| **Name** | **Sequence** | **Supplier** |
| --- | --- | --- |
| Human peripheral blood mononuclear cells | Healthy donors |  |
| Liver-associated mononuclear cells | Healthy donors |  |

- 1. **Biological samples**

| **Description** | **Source** | **Identifier** |
| --- | --- | --- |
|  |  |  |

- 1. **Deposited data**

| **Name of repository** | **Identifier** | **Link** |
| --- | --- | --- |
|  |  |  |

- 1. **Software**

| **Software name** | **Manufacturer** | **Version** |
| --- | --- | --- |
| FlowJo software | BD Biosciences | 10.7.1 |
| Prism | Graphpad | 8 |
| Imaris | Oxford Instruments | 9.1 |

- 1. **Other (*e.g*. drugs, proteins, vectors etc.)**

| Ficoll-Paque PLUS density gradient medium | Cytiva | 17144003 |
| --- | --- | --- |
| FBS | Sigma | F4135 |
| HEPES | Sigma | H0887 |
| Sodium pyruvate | Sigma | S8636 |
| Penicillin/streptomycin | Sigma | P4333 |
| MEM Non-essential Amino Acid Solution (100×) | Sigma | M7145 |
| Clontech Labs Puromycin | Fisher Scientific | NC9359288 |
| RPMI-1640 medium | Sigma | R8005 |
| Dulbecco’s Modified Eagle’s Medium | Sigma | D5796 |
| Geneticin™ Selective Antibiotic (G418 Sulfate) | Gibco | 10131035 |
| Dimethyl sulfoxide | Sigma | D4540 |
| Anti-APC microbeads | Miltenyi | 130-090-855 |
| Anti-PE microbeads | Miltenyi | 130-048-801 |
| Immunocult™-XF T cell Expansion Medium | STEMCELL | 10981 |
| CTS™ Immune Cell Serum Replacement | Thermofisher | A2596101 |
| Normocin™ | Invivogen | ant-nr-1 |
| Animal-Free recombinant IL-2 | Peprotech | AF-200-02 |
| Aim V™ Medium | Thermofisher | 12055091 |
| Human IL-2 IS, premium grade | Miltenyi | 130-097-745 |
| FastDigest XbaI | Thermofisher | FD0684 |
| mMessage mMachine™ T7 ULTRA Transcription Kit | Invitrogen | AMB13455 |
| Cell Line Optimization 4D-Nucleofector^TM^  X Kit | Lonza | V4XC-9064 |
| SF Cell Line 4D-Nucleofector™ X Kit L | Lonza | V4XC-2012 |
| Cell Line Nucleofector™ Kit V | Lonza | VCA-1003 |
| LIVE/DEAD™ Fixable Near-IR Cell Stain Kit | Thermofisher | L34975 |
| BD CellFix (10X concentrate) | BD Biosciences | 340181 |
| BD Cytofix/Cytoperm™ Fixation/Permeabilization Kit | BD Biosciences | 554714 |
| GolgiPlug™ Protein Transport Inhibitor | BD Biosciences | 555029 |
| GolgiStop™ Protein Transport Inhibitor | BD Biosciences | 554724 |
| Recombinant Human IFN-gamma Protein | R&D Systems | 285-IF-100 |
| Recombinant Human TNF-alpha Protein | R&D Systems | 210-TA-005 |
| Phenol red sodium salt | Sigma | P5530 |
| Collagen I (rat tail) | Corning | 354236 |
| Water for Injection (WFI) for Cell Culture | Life Technologies | A1287301 |
| DRAQ7™ | Biolegend | 424001 |
| CellTracker™ Violet BMCQ Dye | Thermofisher | C10094 |
| Recombinant human IL-12p70 | Peprotech | 200-12 |
| Recombinant human IL-18 | MBL | B001-5 |
| Gentamicin | Thermofisher | 15750078 |

- 1. **Please provide the details of the corresponding methods author for the manuscript:**

| Professor Margaret Sällberg Chen, Karolinska Institutet, Sweden.  Email: margaret.chen@ki.se |
| --- |

**2.0 Please confirm for randomised controlled trials all versions of the clinical protocol are included in the submission. These will be published online as supplementary information.**

|  |
| --- |
