# Supplementary material for: Human MAIT cells endowed with HBV specificity are cytotoxic and migrate towards HBV-HCC while retaining antimicrobial functions
Source: JHEP Rep. 2021 Jun 11;3(4):100318. doi: 10.1016/j.jhepr.2021.100318 (PMC8327138; doi:10.1016/j.jhepr.2021.100318)
Supplement: Multimedia component 3 [file mmc3.pdf]

ICMJE Form for Disclosure of Potential Conflicts of Interest

Instructions

The purpose of this form is to provide readers of your manuscript with information about your other interests that could influence how they receive and understand your work. The form is designed to be completed electronically and stored electronically. It contains programming that allows appropriate data display. Each author should submit a separate form and is responsible for the accuracy and completeness of the submitted information. The form is in six parts.

1. Identifying information.

2. The work under consideration for publication.

This section asks for information about the work that you have submitted for publication. The time frame for this reporting is that of the work itself, from the initial conception and planning to the present. The requested information is about resources that you received, either directly or indirectly (via your institution), to enable you to complete the work. Checking "No" means that you did the work without receiving any financial support from any third party -- that is, the work was supported by funds from the same institution that pays your salary and that institution did not receive third-party funds with which to pay you. If you or your institution received funds from a third party to support the work, such as a government granting agency, charitable foundation or commercial sponsor, check "Yes".

3. Relevant financial activities outside the submitted work.

This section asks about your financial relationships with entities in the bio-medical arena that could be perceived to influence, or that give the appearance of potentially influencing, what you wrote in the submitted work. You should disclose interactions with ANY entity that could be considered broadly relevant to the work. For example, if your article is about testing an epidermal growth factor receptor (EGFR) antagonist in lung cancer, you should report all associations with entities pursuing diagnostic or therapeutic strategies in cancer in general, not just in the area of EGFR or lung cancer.

Report all sources of revenue paid (or promised to be paid) directly to you or your institution on your behalf over the 36 months prior to submission of the work. This should include all monies from sources with relevance to the submitted work, not just monies from the entity that sponsored the research. Please note that your interactions with the work's sponsor that are outside the submitted work should also be listed here. If there is any question, it is usually better to disclose a relationship than not to do so.

For grants you have received for work outside the submitted work, you should disclose support ONLY from entities that could be perceived to be affected financially by the published work, such as drug companies, or foundations supported by entities that could be perceived to have a financial stake in the outcome. Public funding sources, such as government agencies, charitable foundations or academic institutions, need not be disclosed. For example, if a government agency sponsored a study in which you have been involved and drugs were provided by a pharmaceutical company, you need only list the pharmaceutical company.

4. Intellectual Property.

This section asks about patents and copyrights, whether pending, issued, licensed and/or receiving royalties.

5. Relationships not covered above.

Use this section to report other relationships or activities that readers could perceive to have influenced, or that give the appearance of potentially influencing, what you wrote in the submitted work.

Definitions.

Entity: government agency, foundation, commercial sponsor, academic institution, etc.
Grant: A grant from an entity, generally [but not always] paid to your organization
Personal Fees: Monies paid to you for services rendered, generally honoraria, royalties, or fees for consulting , lectures, speakers bureaus, expert testimony, employment, or other affiliations
Non-Financial Support: Examples include drugs/equipment supplied by the entity, travel paid by the entity, writing assistance, administrative support, etc**.**

Other: Anything not covered under the previous three boxes
Pending: The patent has been filed but not issued
Issued: The patent has been issued by the agency
Licensed: The patent has been licensed to an entity, whether earning royalties or not
Royalties: Funds are coming in to you or your institution due to your patent

ICMJE Form for Disclosure of Potential Conflicts of Interest

Section 1. Identifying Information

1. Given Name (First Name): Antonio
2. Surname (Last Name): Bertoletti
3. Date: 12-March-2021

4. Are you the corresponding author? ☐ Yes ☑ No

Corresponding Author's Name

5. Manuscript Title
Human MAIT cells endowed with HBV specificity are cytotoxic and migrate towards HBV-HCC while retaining antimicrobial functions

6. Manuscript Identifying Number (if you know it)

Section 2. The Work Under Consideration for Publication

Did you or your institution **at any time** receive payment or services from a third party (government, commercial, private foundation, etc.) for any aspect of the submitted work (including but not limited to grants, data monitoring board, study design, manuscript preparation, statistical analysis, etc.)?

Are there any relevant conflicts of interest? ☐ Yes ☑ No

Section 3. Relevant financial activities outside the submitted work.

Place a check in the appropriate boxes in the table to indicate whether you have financial relationships (regardless of amount of compensation) with entities as described in the instructions. Use one line for each entity; add as many lines as you need by clicking the "Add +" box. You should report relationships that were **present during the 36 months prior to publication**.

Are there any relevant conflicts of interest? ☐ Yes ☑ No

Section 4. Intellectual Property -- Patents & Copyrights

Do you have any patents, whether planned, pending or issued, broadly relevant to the work? ☐ Yes ☑ No

INTERNATIONAL COMMITTEE of MEDICAL JOURNAL EDITORS

ICMJE Form for Disclosure of Potential Conflicts of Interest

Section 5. Relationships not covered above

Are there other relationships or activities that readers could perceive to have influenced, or that give the appearance of potentially influencing, what you wrote in the submitted work?

- [x] Yes, the following relationships/conditions/circumstances are present (explain below):
- [ ] No other relationships/conditions/circumstances that present a potential conflict of interest

Antonio Bertoletti (AB) declares the following relationship with commercial entities developing therapeutics for HBV treatment. He acted as a consultant and served on the advisory boards of Gilead Sciences, MedImmune, Jansseen-Cilag, Abivax, Vir. AB is also a co-founder of LION TCR pte. ltd. a biotech company developing T cell receptors for treatment of virus-related cancers and chronic viral diseases.

At the time of manuscript acceptance, journals will ask authors to confirm and, if necessary, update their disclosure statements. On occasion, journals may ask authors to disclose further information about reported relationships.

Section 6. Disclosure Statement

Based on the above disclosures, this form will automatically generate a disclosure statement, which will appear in the box below.

Dr. Bertoletti reports and
Antonio Bertoletti (AB) declares the following relationship with commercial entities developing therapeutics for HBV treatment. He acted as a consultant and served on the advisory boards of Gilead Sciences, MedImmune, Jansseen-Cilag, Abivax, Vir. AB is also a co-founder of LION TCR pte. ltd. a biotech company developing T cell receptors for treatment of virus-related cancers and chronic viral diseases.
.

Evaluation and Feedback

Please visit http://www.icmje.org/cgi-bin/feedback to provide feedback on your experience with completing this form.

ICMJE DISCLOSURE FORM

Date:2021-03-11
Your Name: Andrea Pavesi
Manuscript Title: Human MAIT cells endowed with HBV specificity are cytotoxic and migrate towards HBV-HCC while retaining antimicrobial functions
Manuscript number (if known): JHEPAT-D-20-02990

In the interest of transparency, we ask you to disclose all relationships/activities/interests listed below that are related to the content of your manuscript. "Related" means any relation with for-profit or not-for-profit third parties whose interests may be affected by the content of the manuscript. Disclosure represents a commitment to transparency and does not necessarily indicate a bias. If you are in doubt about whether to list a relationship/activity/interest, it is preferable that you do so.

The following questions apply to the author's relationships/activities/interests as they relate to the current manuscript only.

The author's relationships/activities/interests should be defined broadly. For example, if your manuscript pertains to the epidemiology of hypertension, you should declare all relationships with manufacturers of antihypertensive medication, even if that medication is not mentioned in the manuscript.

In item #1 below, report all support for the work reported in this manuscript without time limit. For all other items, the time frame for disclosure is the past 36 months.

		**Name all entities with whom you have this relationship or indicate none (add rows as needed)**	**Specifications/Comments (e.g., if payments were made to you or to your institution)**
		**Time frame: Since the initial planning of the work**	
1	All support for the present manuscript (e.g., funding, provision of study materials, medical writing, article processing charges, etc.) **No time limit for this item.**	____None	
		**Time frame: past 36 months**	
2	Grants or contracts from any entity (if not indicated in item #1 above).	AIM Biotech Pte Ltd	My lab have a joint service lab with AIM Biotech.
3	Royalties or licenses	____None	
4	Consulting fees	AIM Biotech Pte Ltd	I'm one of the company consultant .

5	Payment or honoraria for lectures, presentations, speakers bureaus, manuscript writing or educational events	____None	
6	Payment for expert testimony	____None	
7	Support for attending meetings and/or travel	____None	
8	Patents planned, issued or pending	AIM Biotech Pte Ltd	Co-inventor with AIM Biotech
9	Participation on a Data Safety Monitoring Board or Advisory Board	____None	
10	Leadership or fiduciary role in other board, society, committee or advocacy group, paid or unpaid	____None	
11	Stock or stock options	AIM Biotech Pte Ltd	Share holder
12	Receipt of equipment, materials, drugs, medical writing, gifts or other services	____None	
13	Other financial or non-financial interests	____None	

Please place an "X" next to the following statement to indicate your agreement:

___ I certify that I have answered every question and have not altered the wording of any of the questions on this form.

ICMJE DISCLOSURE FORM

Date:2021-03-11
Your Name: TAN Anthony Tanoto
Manuscript Title: Human MAIT cells endowed with HBV specificity are cytotoxic and migrate towards HBV-HCC while retaining antimicrobial functions
Manuscript number (if known): JHEPAT-D-20-02990

In the interest of transparency, we ask you to disclose all relationships/activities/interests listed below that are related to the content of your manuscript. "Related" means any relation with for-profit or not-for-profit third parties whose interests may be affected by the content of the manuscript. Disclosure represents a commitment to transparency and does not necessarily indicate a bias. If you are in doubt about whether to list a relationship/activity/interest, it is preferable that you do so.

The following questions apply to the author's relationships/activities/interests as they relate to the current manuscript only.

The author's relationships/activities/interests should be defined broadly. For example, if your manuscript pertains to the epidemiology of hypertension, you should declare all relationships with manufacturers of antihypertensive medication, even if that medication is not mentioned in the manuscript.

In item #1 below, report all support for the work reported in this manuscript without time limit. For all other items, the time frame for disclosure is the past 36 months.

		**Name all entities with whom you have this relationship or indicate none (add rows as needed)**	**Specifications/Comments (e.g., if payments were made to you or to your institution)**
**Time frame: Since the initial planning of the work**			
1	All support for the present manuscript (e.g., funding, provision of study materials, medical writing, article processing charges, etc.) **No time limit for this item.**	__X__None	
**Time frame: past 36 months**			
2	Grants or contracts from any entity (if not indicated in item #1 above).	_X___None	
3	Royalties or licenses	__X__None	
4	Consulting fees	_X___None	

5	Payment or honoraria for lectures, presentations, speakers bureaus, manuscript writing or educational events	_X___None	
6	Payment for expert testimony	_X___None	
7	Support for attending meetings and/or travel	_X___None	
8	Patents planned, issued or pending	_X___None	
9	Participation on a Data Safety Monitoring Board or Advisory Board	_X___None	
10	Leadership or fiduciary role in other board, society, committee or advocacy group, paid or unpaid	_X___None	
11	Stock or stock options	_X___None	
12	Receipt of equipment, materials, drugs, medical writing, gifts or other services	_X___None	
13	Other financial or non-financial interests	_X___None	

Please place an "X" next to the following statement to indicate your agreement:

_X__ I certify that I have answered every question and have not altered the wording of any of the questions on this form.

ICMJE DISCLOSURE FORM

Date:2021-03-11
Your Name: Haleh Davanian
Manuscript Title: Human MAIT cells endowed with HBV specificity are cytotoxic and migrate towards HBV-HCC while retaining antimicrobial functions
Manuscript number (if known): JHEPAT-D-20-02990

In the interest of transparency, we ask you to disclose all relationships/activities/interests listed below that are related to the content of your manuscript. "Related" means any relation with for-profit or not-for-profit third parties whose interests may be affected by the content of the manuscript. Disclosure represents a commitment to transparency and does not necessarily indicate a bias. If you are in doubt about whether to list a relationship/activity/interest, it is preferable that you do so.

The following questions apply to the author's relationships/activities/interests as they relate to the current manuscript only.

The author's relationships/activities/interests should be defined broadly. For example, if your manuscript pertains to the epidemiology of hypertension, you should declare all relationships with manufacturers of antihypertensive medication, even if that medication is not mentioned in the manuscript.

In item #1 below, report all support for the work reported in this manuscript without time limit. For all other items, the time frame for disclosure is the past 36 months.

		Name all entities with whom you have this relationship or indicate none (add rows as needed)	Specifications/Comments (e.g., if payments were made to you or to your institution)
**Time frame: Since the initial planning of the work**			
1	All support for the present manuscript (e.g., funding, provision of study materials, medical writing, article processing charges, etc.) **No time limit for this item.**	____None	
**Time frame: past 36 months**			
2	Grants or contracts from any entity (if not indicated in item #1 above).	____None	
3	Royalties or licenses	____None	
4	Consulting fees	____None	

5	Payment or honoraria for lectures, presentations, speakers bureaus, manuscript writing or educational events	____None	
6	Payment for expert testimony	____None	
7	Support for attending meetings and/or travel	____None	
8	Patents planned, issued or pending	____None	
9	Participation on a Data Safety Monitoring Board or Advisory Board	____None	
10	Leadership or fiduciary role in other board, society, committee or advocacy group, paid or unpaid	____None	
11	Stock or stock options	____None	
12	Receipt of equipment, materials, drugs, medical writing, gifts or other services	____None	
13	Other financial or non-financial interests	____None	

Please place an "X" next to the following statement to indicate your agreement:

_X_ I certify that I have answered every question and have not altered the wording of any of the questions on this form.

ICMJE DISCLOSURE FORM

Date:2021-03-11
Your Name: Johan Sandberg
Manuscript Title: Human MAIT cells endowed with HBV specificity are cytotoxic and migrate towards HBV-HCC while retaining antimicrobial functions
Manuscript number (if known): JHEPAT-D-20-02990

In the interest of transparency, we ask you to disclose all relationships/activities/interests listed below that are related to the content of your manuscript. "Related" means any relation with for-profit or not-for-profit third parties whose interests may be affected by the content of the manuscript. Disclosure represents a commitment to transparency and does not necessarily indicate a bias. If you are in doubt about whether to list a relationship/activity/interest, it is preferable that you do so.

The following questions apply to the author's relationships/activities/interests as they relate to the current manuscript only.

The author's relationships/activities/interests should be defined broadly. For example, if your manuscript pertains to the epidemiology of hypertension, you should declare all relationships with manufacturers of antihypertensive medication, even if that medication is not mentioned in the manuscript.

In item #1 below, report all support for the work reported in this manuscript without time limit. For all other items, the time frame for disclosure is the past 36 months.

		**Name all entities with whom you have this relationship or indicate none (add rows as needed)**	**Specifications/Comments (e.g., if payments were made to you or to your institution)**
**Time frame: Since the initial planning of the work**			
1	All support for the present manuscript (e.g., funding, provision of study materials, medical writing, article processing charges, etc.) **No time limit for this item.**	Janssen Pharmaceutica	Project funding to my institution which contributed in part to the work in this manuscript
**Time frame: past 36 months**			
2	Grants or contracts from any entity (if not indicated in item #1 above).	____None	
3	Royalties or licenses	____None	
4	Consulting fees	____None	

5	Payment or honoraria for lectures, presentations, speakers bureaus, manuscript writing or educational events	____None	
6	Payment for expert testimony	____None	
7	Support for attending meetings and/or travel	____None	
8	Patents planned, issued or pending	____None	
9	Participation on a Data Safety Monitoring Board or Advisory Board	____None	
10	Leadership or fiduciary role in other board, society, committee or advocacy group, paid or unpaid	____None	
11	Stock or stock options	____None	
12	Receipt of equipment, materials, drugs, medical writing, gifts or other services	____None	
13	Other financial or non-financial interests	____None	

Please place an "X" next to the following statement to indicate your agreement:

__X_ I certify that I have answered every question and have not altered the wording of any of the questions on this form.

ICMJE DISCLOSURE FORM

Date:2021-03-11
Your Name: Katie Healy
Manuscript Title: Human MAIT cells endowed with HBV specificity are cytotoxic and migrate towards HBV-HCC while retaining antimicrobial functions
Manuscript number (if known): JHEPAT-D-20-02990

In the interest of transparency, we ask you to disclose all relationships/activities/interests listed below that are related to the content of your manuscript. "Related" means any relation with for-profit or not-for-profit third parties whose interests may be affected by the content of the manuscript. Disclosure represents a commitment to transparency and does not necessarily indicate a bias. If you are in doubt about whether to list a relationship/activity/interest, it is preferable that you do so.

The following questions apply to the author's relationships/activities/interests as they relate to the current manuscript only.

The author's relationships/activities/interests should be defined broadly. For example, if your manuscript pertains to the epidemiology of hypertension, you should declare all relationships with manufacturers of antihypertensive medication, even if that medication is not mentioned in the manuscript.

In item #1 below, report all support for the work reported in this manuscript without time limit. For all other items, the time frame for disclosure is the past 36 months.

		**Name all entities with whom you have this relationship or indicate none (add rows as needed)**	**Specifications/Comments (e.g., if payments were made to you or to your institution)**
**Time frame: Since the initial planning of the work**			
1	All support for the present manuscript (e.g., funding, provision of study materials, medical writing, article processing charges, etc.) **No time limit for this item.**	Erik and Edith Fernström Foundation	Scholarship paid to me through my institution (Karolinska Institutet) to cover my expenses while doing a research visit in our collaborators lab.
**Time frame: past 36 months**			
2	Grants or contracts from any entity (if not indicated in item #1 above).	X___None	
3	Royalties or licenses	X____None	

4	Consulting fees	X____None	
5	Payment or honoraria for lectures, presentations, speakers bureaus, manuscript writing or educational events	X____None	
6	Payment for expert testimony	X____None	
7	Support for attending meetings and/or travel	X____None	
8	Patents planned, issued or pending	X____None	
9	Participation on a Data Safety Monitoring Board or Advisory Board	X____None	
10	Leadership or fiduciary role in other board, society, committee or advocacy group, paid or unpaid	X____None	
11	Stock or stock options	X____None	
12	Receipt of equipment, materials, drugs, medical writing, gifts or other services	X____None	
13	Other financial or non-financial interests	X____None	

Please place an "X" next to the following statement to indicate your agreement:

__X_ I certify that I have answered every question and have not altered the wording of any of the questions on this form.

ICMJE DISCLOSURE FORM

Date:2021-03-11
Your Name: Michał J. Sobkowiak
Manuscript Title: Human MAIT cells endowed with HBV specificity are cytotoxic and migrate towards HBV-HCC while retaining antimicrobial functions
Manuscript number (if known): JHEPAT-D-20-02990

In the interest of transparency, we ask you to disclose all relationships/activities/interests listed below that are related to the content of your manuscript. "Related" means any relation with for-profit or not-for-profit third parties whose interests may be affected by the content of the manuscript. Disclosure represents a commitment to transparency and does not necessarily indicate a bias. If you are in doubt about whether to list a relationship/activity/interest, it is preferable that you do so.

The following questions apply to the author's relationships/activities/interests as they relate to the current manuscript only.

The author's relationships/activities/interests should be defined broadly. For example, if your manuscript pertains to the epidemiology of hypertension, you should declare all relationships with manufacturers of antihypertensive medication, even if that medication is not mentioned in the manuscript.

In item #1 below, report all support for the work reported in this manuscript without time limit. For all other items, the time frame for disclosure is the past 36 months.

		**Name all entities with whom you have this relationship or indicate none (add rows as needed)**	**Specifications/Comments (e.g., if payments were made to you or to your institution)**
		**Time frame: Since the initial planning of the work**	
1	All support for the present manuscript (e.g., funding, provision of study materials, medical writing, article processing charges, etc.) **No time limit for this item.**	__X__None	
		**Time frame: past 36 months**	
2	Grants or contracts from any entity (if not indicated in item #1 above).	__X__None	
3	Royalties or licenses	__X__None	
4	Consulting fees	__X__None	

5	Payment or honoraria for lectures, presentations, speakers bureaus, manuscript writing or educational events	__X__None	
6	Payment for expert testimony	__X__None	
7	Support for attending meetings and/or travel	__X__None	
8	Patents planned, issued or pending	__X__None	
9	Participation on a Data Safety Monitoring Board or Advisory Board	__X__None	
10	Leadership or fiduciary role in other board, society, committee or advocacy group, paid or unpaid	__X__None	
11	Stock or stock options	__X__None	
12	Receipt of equipment, materials, drugs, medical writing, gifts or other services	__X__None	
13	Other financial or non-financial interests	__X__None	

Please place an "X" next to the following statement to indicate your agreement:

_X__ I certify that I have answered every question and have not altered the wording of any of the questions on this form.

ICMJE DISCLOSURE FORM

Date:2021-03-11
Your Name: Margaret Sällberg Chen
Manuscript Title: Human MAIT cells endowed with HBV specificity are cytotoxic and migrate towards HBV-HCC while retaining antimicrobial functions
Manuscript number (if known): JHEPAT-D-20-02990

In the interest of transparency, we ask you to disclose all relationships/activities/interests listed below that are related to the content of your manuscript. "Related" means any relation with for-profit or not-for-profit third parties whose interests may be affected by the content of the manuscript. Disclosure represents a commitment to transparency and does not necessarily indicate a bias. If you are in doubt about whether to list a relationship/activity/interest, it is preferable that you do so.

The following questions apply to the author's relationships/activities/interests as they relate to the current manuscript only.

The author's relationships/activities/interests should be defined broadly. For example, if your manuscript pertains to the epidemiology of hypertension, you should declare all relationships with manufacturers of antihypertensive medication, even if that medication is not mentioned in the manuscript.

In item #1 below, report all support for the work reported in this manuscript without time limit. For all other items, the time frame for disclosure is the past 36 months.

		Name all entities with whom you have this relationship or indicate none (add rows as needed)	Specifications/Comments (e.g., if payments were made to you or to your institution)
		**Time frame: Since the initial planning of the work**	
1	All support for the present manuscript (e.g., funding, provision of study materials, medical writing, article processing charges, etc.) **No time limit for this item.**	Swedish Cancer Society	To my research group at Karolinska institutet (I am PI)
		Karolinska Institutet funding for doctoral students (KID-funding)	To my research group at Karolinska institutet (I am PI)
		KI – Johnson & Johnson Innovation/Janssen grant	To my research group at Karolinska institutet (I am co-PI)
		**Time frame: past 36 months**	
2	Grants or contracts from any entity (if not indicated in item #1 above).	_X___None	
3	Royalties or licenses	_X__None	

4	Consulting fees	_X_None	
5	Payment or honoraria for lectures, presentations, speakers bureaus, manuscript writing or educational events	X_None	
6	Payment for expert testimony	X_None	
7	Support for attending meetings and/or travel	_X_None	
8	Patents planned, issued or pending	_X_None	
9	Participation on a Data Safety Monitoring Board or Advisory Board	X_None	
10	Leadership or fiduciary role in other board, society, committee or advocacy group, paid or unpaid	_X_None	
11	Stock or stock options	_X__None	
12	Receipt of equipment, materials, drugs, medical writing, gifts or other services	__X__None	
13	Other financial or non-financial interests	_X___None	

Please place an "X" next to the following statement to indicate your agreement:

_X_ I certify that I have answered every question and have not altered the wording of any of the questions on this form.

ICMJE DISCLOSURE FORM

Date:2021-03-11
Your Name:
Manuscript Title: Human MAIT cells endowed with HBV specificity are cytotoxic and migrate towards HBV-HCC while retaining antimicrobial functions
Manuscript number (if known): JHEPAT-D-20-02990

In the interest of transparency, we ask you to disclose all relationships/activities/interests listed below that are related to the content of your manuscript. "Related" means any relation with for-profit or not-for-profit third parties whose interests may be affected by the content of the manuscript. Disclosure represents a commitment to transparency and does not necessarily indicate a bias. If you are in doubt about whether to list a relationship/activity/interest, it is preferable that you do so.

The following questions apply to the author's relationships/activities/interests as they relate to the current manuscript only.

The author's relationships/activities/interests should be defined broadly. For example, if your manuscript pertains to the epidemiology of hypertension, you should declare all relationships with manufacturers of antihypertensive medication, even if that medication is not mentioned in the manuscript.

In item #1 below, report all support for the work reported in this manuscript without time limit. For all other items, the time frame for disclosure is the past 36 months.

		**Name all entities with whom you have this relationship or indicate none (add rows as needed)**	**Specifications/Comments (e.g., if payments were made to you or to your institution)**
		**Time frame: Since the initial planning of the work**	
1	All support for the present manuscript (e.g., funding, provision of study materials, medical writing, article processing charges, etc.) **No time limit for this item.**	X None	
		**Time frame: past 36 months**	
2	Grants or contracts from any entity (if not indicated in item #1 above).	X None	
3	Royalties or licenses	X None	
4	Consulting fees	____None	MSD, Biogen

5	Payment or honoraria for lectures, presentations, speakers bureaus, manuscript writing or educational events	X None	
6	Payment for expert testimony	X None	
7	Support for attending meetings and/or travel	X None	
8	Patents planned, issued or pending	X None	
9	Participation on a Data Safety Monitoring Board or Advisory Board	X None	
10	Leadership or fiduciary role in other board, society, committee or advocacy group, paid or unpaid	X None	
11	Stock or stock options	X None	
12	Receipt of equipment, materials, drugs, medical writing, gifts or other services	X None	
13	Other financial or non-financial interests	X None	

Please place an "X" next to the following statement to indicate your agreement:

X **I certify that I have answered every question and have not altered the wording of any of the questions on this form.**

SOO ALEMAN

ICMJE DISCLOSURE FORM

Date:2021-03-11
Your Name:
Manuscript Title: Human MAIT cells endowed with HBV specificity are cytotoxic and migrate towards HBV-HCC while retaining antimicrobial functions
Manuscript number (if known): JHEPAT-D-20-02990

In the interest of transparency, we ask you to disclose all relationships/activities/interests listed below that are related to the content of your manuscript. "Related" means any relation with for-profit or not-for-profit third parties whose interests may be affected by the content of the manuscript. Disclosure represents a commitment to transparency and does not necessarily indicate a bias. If you are in doubt about whether to list a relationship/activity/interest, it is preferable that you do so.

The following questions apply to the author's relationships/activities/interests as they relate to the <u>current manuscript only</u>.

The author's relationships/activities/interests should be <u>defined broadly</u>. For example, if your manuscript pertains to the epidemiology of hypertension, you should declare all relationships with manufacturers of antihypertensive medication, even if that medication is not mentioned in the manuscript.

In item #1 below, report all support for the work reported in this manuscript without time limit. For all other items, the time frame for disclosure is the past 36 months.

		**Name all entities with whom you have this relationship or indicate none (add rows as needed)**	**Specifications/Comments (e.g., if payments were made to you or to your institution)**
		**Time frame: Since the initial planning of the work**	
1	All support for the present manuscript (e.g., funding, provision of study materials, medical writing, article processing charges, etc.) **No time limit for this item.**	____None	
		Susanne E Reinsbach	Knut and Alice Wallenberg Foundation as part of the National Bioinformatics Infrastructure Sweden at SciLifeLab
		**Time frame: past 36 months**	
2	Grants or contracts from any entity (if not indicated in item #1 above).	__x__None	
3	Royalties or licenses	__x__None	

4	Consulting fees	__x__None	
5	Payment or honoraria for lectures, presentations, speakers bureaus, manuscript writing or educational events	__x__None	
6	Payment for expert testimony	__x__None	
7	Support for attending meetings and/or travel	__x__None	
8	Patents planned, issued or pending	__x__None	
9	Participation on a Data Safety Monitoring Board or Advisory Board	__x__None	
10	Leadership or fiduciary role in other board, society, committee or advocacy group, paid or unpaid	__x__None	
11	Stock or stock options	__x__None	
12	Receipt of equipment, materials, drugs, medical writing, gifts or other services	__x__None	
13	Other financial or non-financial interests	__x__None	

Please place an "X" next to the following statement to indicate your agreement:

__x_ I certify that I have answered every question and have not altered the wording of any of the questions on this form.

ICMJE DISCLOSURE FORM

Date:2021-03-11
Your Name: Tiphaine Parrot
Manuscript Title: Human MAIT cells endowed with HBV specificity are cytotoxic and migrate towards HBV-HCC while retaining antimicrobial functions
Manuscript number (if known): JHEPAT-D-20-02990

In the interest of transparency, we ask you to disclose all relationships/activities/interests listed below that are related to the content of your manuscript. "Related" means any relation with for-profit or not-for-profit third parties whose interests may be affected by the content of the manuscript. Disclosure represents a commitment to transparency and does not necessarily indicate a bias. If you are in doubt about whether to list a relationship/activity/interest, it is preferable that you do so.

The following questions apply to the author's relationships/activities/interests as they relate to the current manuscript only.

The author's relationships/activities/interests should be defined broadly. For example, if your manuscript pertains to the epidemiology of hypertension, you should declare all relationships with manufacturers of antihypertensive medication, even if that medication is not mentioned in the manuscript.

In item #1 below, report all support for the work reported in this manuscript without time limit. For all other items, the time frame for disclosure is the past 36 months.

		**Name all entities with whom you have this relationship or indicate none (add rows as needed)**	**Specifications/Comments (e.g., if payments were made to you or to your institution)**
**Time frame: Since the initial planning of the work**			
1	All support for the present manuscript (e.g., funding, provision of study materials, medical writing, article processing charges, etc.) **No time limit for this item.**	None	
**Time frame: past 36 months**			
2	Grants or contracts from any entity (if not indicated in item #1 above).	None	
3	Royalties or licenses	None	
4	Consulting fees	None	

5	Payment or honoraria for lectures, presentations, speakers bureaus, manuscript writing or educational events	None	
6	Payment for expert testimony	None	
7	Support for attending meetings and/or travel	None	
8	Patents planned, issued or pending	None	
9	Participation on a Data Safety Monitoring Board or Advisory Board	None	
10	Leadership or fiduciary role in other board, society, committee or advocacy group, paid or unpaid	None	
11	Stock or stock options	None	
12	Receipt of equipment, materials, drugs, medical writing, gifts or other services	None	
13	Other financial or non-financial interests	None	

Please place an "X" next to the following statement to indicate your agreement:

__X_ I certify that I have answered every question and have not altered the wording of any of the questions on this form.
